# Metal-organic framework crystal-glass composites

**DOI:** 10.1038/s41467-019-10470-z

**Published:** 2019-06-12

**Authors:** Jingwei Hou, Christopher W. Ashling, Sean M. Collins, Andraž Krajnc, Chao Zhou, Louis Longley, Duncan N. Johnstone, Philip A. Chater, Shichun Li, Marie-Vanessa Coulet, Philip L. Llewellyn, François-Xavier Coudert, David A. Keen, Paul A. Midgley, Gregor Mali, Vicki Chen, Thomas D. Bennett

**Affiliations:** 1 Department of Materials Science and Metallurgy, University of Cambridge, Cambridge, CB3 0FS UK; 2 Department of Inorganic Chemistry and Technology, National Institute of Chemistry, 1001 Ljubljana, Slovenia; 3 Diamond Light Source Ltd., Diamond House, Harwell Science & Innovation Campus, Didcot, Oxfordshire OX11 0DE UK; 4 Institute of Chemical Materials, China Academy of Engineering Physics, Mianyang, 621900 China; 5 Aix-Marseille Univ, CNRS, MADIREL (UMR 7246), Centre de St. Jérôme, 13397 Marseille cedex 20, France; 6 Chimie ParisTech, PSL University, CNRS, Institut de Recherche de Chimie Paris, 75005 Paris, France; 7 ISIS Facility, Rutherford Appleton Laboratory, Harwell Campus, Didcot, Oxon OX11 0QX UK; 8 School of Chemical Engineering, University of New South Wales, Sydney, NSW 2052 Australia; 9 School of Chemical Engineering, University of Queensland, St. Lucia, QLD 4072 Australia

**Keywords:** Metal-organic frameworks, Porous materials, Organic-inorganic nanostructures, Glasses

## Abstract

The majority of research into metal-organic frameworks (MOFs) focuses on their crystalline nature. Recent research has revealed solid-liquid transitions within the family, which we use here to create a class of functional, stable and porous composite materials. Described herein is the design, synthesis, and characterisation of MOF crystal-glass composites, formed by dispersing crystalline MOFs within a MOF-glass matrix. The coordinative bonding and chemical structure of a MIL-53 crystalline phase are preserved within the ZIF-62 glass matrix. Whilst separated phases, the interfacial interactions between the closely contacted microdomains improve the mechanical properties of the composite glass. More significantly, the high temperature open pore phase of MIL-53, which spontaneously transforms to a narrow pore upon cooling in the presence of water, is stabilised at room temperature in the crystal-glass composite. This leads to a significant improvement of CO_2_ adsorption capacity.

## Abstract (web summary)

The formation of composite materials has been widely exploited to alter the chemical and physical properties of their components. Here the authors form metal–organic framework (MOF) crystal–glass composites in which a MOF glass matrix stabilises the open pore structure of MIL-53, leading to enhanced CO_2_ adsorption.

## Introduction

Metal–organic frameworks (MOFs) are a class of hybrid materials, composed of metal nodes and coordinating organic linkers. The arrangement of these components in highly regular motifs often leads to materials exhibiting ultra-high surface areas^1^. Applications are therefore proposed which utilise this porosity for reversible host–guest behaviour, for example, in gas storage, catalysis and drug delivery^2–6^. Several MOF-based products have been commercialised, such as for delaying the over-ripening of fruit, and for harmful gas storage (e.g., PH_3_) within the semiconductor industry^7^.

The main body of MOF research typically focuses on the discovery of new materials and expanding the library of available crystalline MOFs, which currently stands at over 70,000^8^. Attempts have been made to develop existing MOFs and explore new applications using known functionalities, and introducing flexibility, defects and stimuli responsive behaviour^9,10^. Whilst crystalline MOFs have shown exceptional properties, a number of industrial practicability issues remain. One barrier is the inherent difficulties in processing and shaping MOF microcrystalline powders into mechanically robust macroscale morphologies^11,12^. Conventionally, high pressure pelletisation or binders are used in the shaping of MOF powders but these treatments have been shown to significantly decrease material efficacy^13^.

The formation of composites by combining MOFs with more processable materials such as polymers, not only engages with the theme of new materials discovery, but also offers solutions to the aforementioned problems in manufacturing robust bulk structures. These include core–shell structures, in which a MOF outer layer is grown on an inner sphere of another material^14,15^. Amongst these macroscale architectures, membranes and thin films are particularly important given the requirements for continuous, defect free coverage and flexibility under pressure^16^. Mixed matrix membranes (MMMs) are a prototypical case of such materials^17^. Here, a crystalline MOF filler is typically dispersed in an organic polymer^18^. The disordered nature of the polymeric organic component within MMMs provides both structural stability and facilitates shaping. Significant penalties are incurred however, including pore blocking by the matrix, aggregation of the filler and poor adhesion between the two components, which prevents high loading capacities^19^. Therefore, the synthesis and characterisation of composite MOF materials without these disadvantages is of great importance to bridge the divide between advanced MOF material synthesis and practical device fabrication.

Structural disorder is an emerging topic in the MOF field. In particular, solid–liquid transitions upon heating in both the phosphonate coordination polymer and the zeolitic imidazolate framework (ZIF) families are of interest^20,21^. The latter family contains tetrahedral metal ions, linked by imidazolate (Im – C_3_H_3_N_2_^−^) derived bidentate ligands. Studies of the ZIF-zni [Zn(Im)_2_] structure show that at *ca*. 550 °C, rapid dissociation-association of the imidazolate linker around Zn^2+^ centres occurs, leading to formation of a viscous liquid of identical chemical composition^22^.

The porous glasses formed upon quenching these high temperature liquid ZIFs has been modelled by continuous random network topologies, analogous to amorphous silica. Here, we exploit the disordered MOF state as an analogue for the organic matrix component of MOF–organic composites and create a class of materials comprising crystalline MOFs embedded in a host MOF–glass matrix. These composites, which we term MOF crystal–glass composites (CGCs), might be expected to display better interfacial binding between filler and matrix components than their MMM counterparts, given their greater degree of chemical compatibility. They may also, importantly, exhibit a diverse array of mechanical and structural properties different to those of either parent phase.

ZIF-62 [Zn(Im)_1.75_(bIm)_0.25_] (bIm = benzimidazolate, C_7_H_5_N_2_^−^) was selected as the MOF–glass matrix due to a relatively low temperature of melting (*T*_m_ = 430 °C) and a large temperature range over which the resultant liquid is stable before decomposing (at *ca*. 550 °C). The glass, here referred to as *a*_g_ZIF-62, which is formed upon cooling the ZIF-62 liquid, is also extremely stable against crystallisation, which is ascribed to the high viscosity of the liquid phase^23^.

**Fig. 1:**
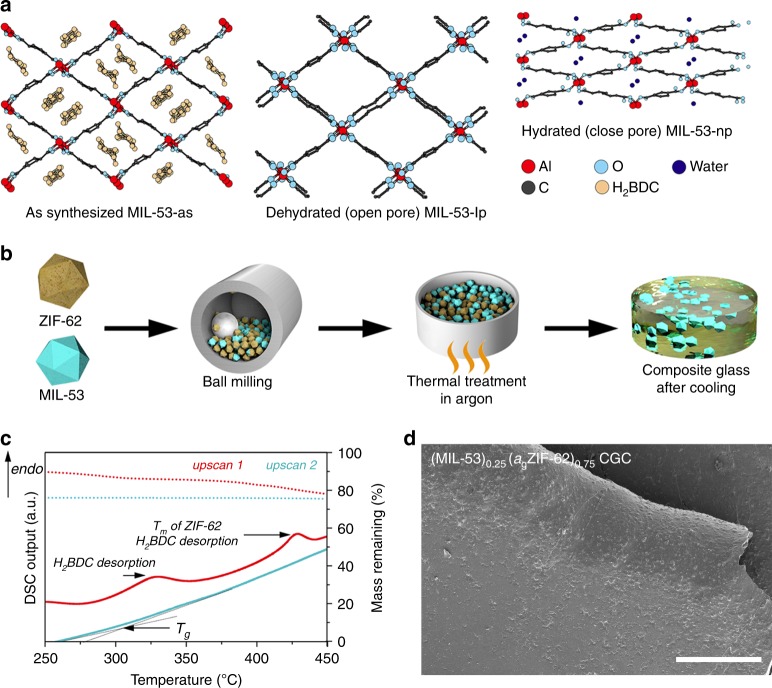
Fabrication of crystal-glass composites. **a** Schematic diagram of the crystal structure of different MIL-53 forms^28^. **b** Schematic diagram of the CGC fabrication process. **c** Thermogravimetric (dotted line) and enthalpic responses (solid line) of (MIL-53)(ZIF-62)(25/75) mixture on both the first (red) and second (blue) DSC heating upscans at 10 °C min^−1^. Melting temperature (offset temperature of the melting peak, *T*_m_), glass transition temperature (onset temperature of glass transition peak, *T*_g_) and H_2_BDC desorption are indicated. **d** SEM image of the (MIL-53)_0.25_(*a*_g_ZIF-62)_0.75_ CGC. Scale bar is 200 µm

The two key requirements for the crystalline component in such a composite, are that the temperature of decomposition (*T*_d_) should exceed the glass-forming matrix *T*_m_, and that the chemical (in)compatibility is such that no flux melting occurs^24^. The two frameworks we chose, MIL-53(Al) [Al(OH)(O_2_C-C_6_H_4_-CO_2_)] and UiO-66 [Zr_6_O_4_(OH)_4_(O_2_C-C_6_H_4_-CO_2_)_6_] both fulfil these criteria^25,26^. MIL-53(Al) is an aluminium 1,4-benzenedicarboxylate (BDC) based MOF (referred to as MIL-53 hereafter), with a 3D framework structure built with trans chains of corner-sharing AlO_4_(OH)_2_ octahedra^27,28^. The as-synthesised (MIL-53-as) structure contains unreacted H_2_BDC within the framework. The removal of these guest molecules by thermal treatment leads to an open-pore structure (MIL-53-lp)^29^. Physisorption of water by MIL-53-lp causes a transition to a closed pore structure (MIL-53-np), due to formation of framework-guest (water molecule) interactions (Fig. 1a). UiO-66, on the other hand, consists of Zr-centred secondary building units connected to (in a perfect crystal) 12 BDC linkers. The crystal structure of UiO-66 is rigid with high thermal and mechanical stability, due to the strong Zr–O bonds and a close-packed structure^30^.

This paper describes the fabrication and characterisation of two MOF crystal-glass composites (CGCs), comprised of MIL-53 and UiO-66 dispersed in *a*_g_ZIF-62. A suitable preparation technique is established, and the intra-domain structures and inter-domain interface interactions of these materials are reported. An insight into the composite microstructure is built up through combined differential scanning calorimetry—thermogravimetric analysis (DSC-TGA), in situ synchrotron powder X-ray diffraction (XRD), 3D X-ray energy dispersive spectroscopy (EDS) tomography, scanning electron diffraction (SED), X-ray total scattering and pair distribution function (PDF) analysis and magic angle spinning nuclear magnetic resonance (NMR) experiments. The functional characteristics of the CGCs are subsequently explored through measurement of their mechanical properties and analysis of their gas adsorption behaviour.

## Results

### MOF CGC fabrication

Pure samples of ZIF-62, MIL-53 and UiO-66 were synthesised (see Methods, Supplementary Fig. 1a–c) and DSC-TGA experiments were carried out under an inert Ar atmosphere to confirm the expected thermal behaviour (Supplementary Fig. 2). The TGA trace of ZIF-62 was featureless between the initial desolvation at *ca*. 250 °C and decomposition at *ca*. 550 °C. The simultaneous DSC measurement, however, showed an endothermic response attributable to a solid–liquid transition (melting) at 435 °C. The corresponding experiments for MIL-53 suggested a two-stage weight loss process during heating. These are consistent with the removal of surface-adsorbed and encapsulated H_2_BDC within the large-breathing framework^28^. Subsequent DSC traces of MIL-53 samples heated to 450 °C and cooled back to room temperature, were featureless. For the pure UiO-66 sample, features ascribed to desolvation below 200 °C, dehydroxlyation of the inorganic cluster at 300 °C and thermal degradation at ~460 °C were observed, in accordance with published literature results^30^.

A fabrication process for the MOF CGC was developed (Fig. 1b). ZIF-62 and MIL-53 (or UiO-66) were ball-milled together (30 Hz, 5 min) to homogenise the mixture (Supplementary Fig. 3). No significant change in crystalline structure was observed (Supplementary Fig. 1d, e). Framework activation was not performed prior to ball-milling, as the presence of solvent within MOFs has been observed to stabilise against shear-induced collapse^31^.

The mixtures of MIL-53 and ZIF-62 after ball milling are referred to as (MIL-53)(ZIF-62)(X/Y), where *X* and *Y* are percentage by mass of each component. For example, a 25 wt% sample of crystalline MIL-53 and 75 wt% crystalline ZIF-62 sample is referred to as (MIL-53)(ZIF-62)(25/75). A series of (MIL-53)(ZIF-62)(*X*/*Y*) samples were then heated in flowing Ar to 450 °C, i.e., above the melting temperature of ZIF-62, but below that of the thermal decomposition temperature of MIL-53. The samples were held at 450 °C for 10 min and then cooled back to room temperature under Ar protection, over a period of approximately 90 min. In keeping with prior terminology on blended ZIFs, the resultant CGCs obtained upon cooling are referred to as (MIL-53)_*X*_(*a*_g_ZIF-62)_*Y*_^32^.

The first TGA trace of (MIL-53)(ZIF-62)(25/75) had a two-stage weight loss, consistent with the desorption of H_2_BDC from MIL-53 (Fig. 1c). The accompanying DSC indicated a broad endotherm at the expected melting temperature of ZIF-62 (Supplementary Figs. 2 and 4), which is expected given the overlapping proximity in temperature ranges of both ZIF-62 melting and MIL-53 desorption phenomena. DSC-TGA heating experiments of the formed (MIL-53)_0.25_(*a*_g_ZIF-62)_0.75_ CGC demonstrated a glass transition, *T*_g_, of ~310 °C (Fig. 1c) and the melted samples, when cooled under Ar protection, appeared glassy with significant morphological changes due to vitrification. Optically transparent glasses could be obtained by clamping the crystalline powder mix between two glass sides during heating (Supplementary Fig. 5). Scanning electron microscopy (SEM) performed on (MIL-53)_0.25_(*a*_g_ZIF-62)_0.75_ (Fig. 1d) suggested good interfacial compatibility for the two different phases within the composite glass. Coherent and continuous composite morphologies were also obtained at higher MIL-53 loadings (Supplementary Fig. 6), up to 75 wt%. Ambient temperature powder XRD data from the CGCs showed that the Bragg scattering from the MIL-53-lp phase was preserved in all cases. This was also true for a sample of (MIL-53)_0.25_(*a*_g_ZIF-62)_0.75_ that was heat treated at 450 °C for 3 h (Supplementary Fig. 7).

An identical methodology was used in attempts to fabricate an equivalent MOF CGC using UiO-66. Broadened melting peaks from ZIF-62 were observed in the TGA-DSC (Supplementary Fig. 8), which we ascribe to the simultaneous onset of UiO-66 decomposition. For the second upscan, the glass transition temperature (*T*_g_) of ZIF-62 overlapped with the dehydroxlyation of the UiO-66 inorganic cluster in the same temperature region, obfuscating exact *T*_g_ determination. Bragg diffraction from the UiO-66 component within the recovered composite product was observed after isothermal treatment of the sample for 10 min at 450 °C. No Bragg diffraction was, however, observed in recovered samples held for 3 h at 450 °C, due to the gradual decomposition of the crystal phase (Supplementary Fig. 9). SEM imaging of the samples with different UiO-66 loadings, held for 10 min at 450 °C, demonstrated the formation of macroporous CGC structures upon heating. We attribute this to partial decomposition (Supplementary Fig. 10), though the nature of this lies outside of the scope of the current publication.

### Component integrity and distribution

The melting and structural collapse of ZIF-62 was further investigated by in situ synchrotron variable temperature powder diffraction. Bragg diffraction from ZIF-62 became weaker after the removal of ZIF-62 solvent, and disappeared completely above the *T*_m_ of 435 °C. The emergence of diffuse scattering at *q* ~0.9–1.2 Å^−1^ at this temperature indicated melting, consistent with prior literature (Supplementary Fig. 11a, b)^32^.

**Fig. 2:**
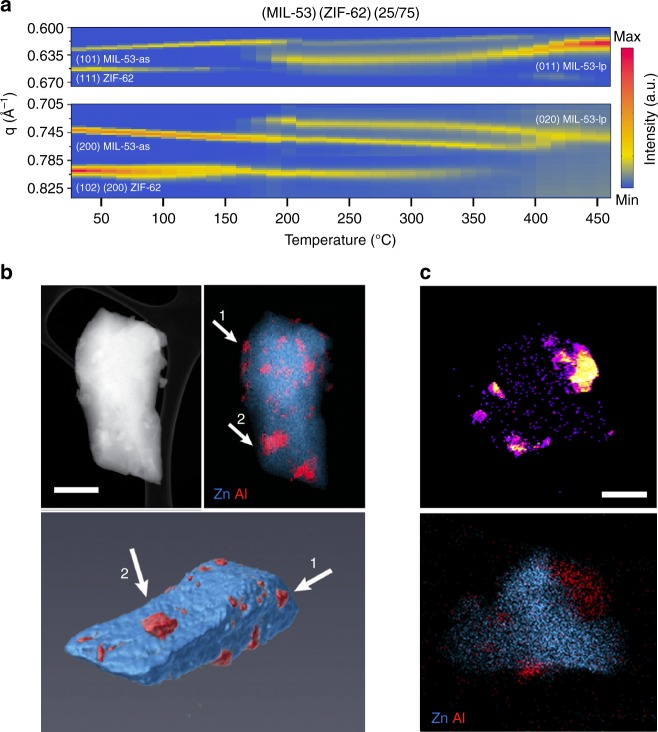
Distribution of crystalline/amorphous phases with the (MIL-53)_0.25_(a_g_ZIF-62)_0.75_ CGC. **a** Contour plots of in situ synchrotron powder diffraction data measured during the thermal treatment process of (MIL-53)(ZIF-62)(25/75) (10 °C min^−1^). The Bragg peak hkl indices are marked for ZIF-62, MIL-53-as and MIL-53-lp. **b** Three-dimensional tomography and the corresponding STEM-EDS mapping of (MIL-53)_0.25_(*a*_g_ZIF-62)_0.75_ CGC. White arrows highlight the region of Al in both 2D and their corresponding 3D images. Scale bar is 400 nm. **c** Scanning electron diffraction (SED) mapping and the corresponding STEM-EDS mapping of the composite glass. Scale bar is 200 nm

Identical experiments were performed on (MIL-53)(ZIF-62)(25/75) and (UiO-66)(ZIF-62)(25/75). As in the experiment performed on pure ZIF-62, Bragg diffraction from ZIF-62 ceased at 435 °C (Fig. 2a and Supplementary Fig. 11c–f). For the (MIL-53)(ZIF-62)(25/75) sample, although an abrupt change at *ca.* 160 °C is observed in the diffraction patterns, indicating a transition between the initial MIL-53-as and the final MIL-53-lp phase, both phases coexist for a further ca. 260 °C. For example, above ca. 160 °C, the MIL-53-lp (011) Bragg peak at 0.62–0.65 Å^−1^ grows in intensity, accompanied by a reduction in intensity of the MIL-53-as (101) Bragg peak at 0.61–0.62 Å^−1^^33^. Peaks from both phases remain until 420 °C, when only Bragg peaks arising from MIL-53-lp are observed. We ascribe this broad transition to the constant heating rate used and the need to perform the experiment in a sealed capillary under Ar.

Unit cell parameters for the MIL-53 and ZIF-62 components were refined by fitting each diffraction pattern in Supplementary Fig. 11 using Pawley fitting across the temperature range (Supplementary Fig. 12, Supplementary Table 1). A large increase in cell volume for MIL-53-lp was noted above 350 °C, i.e., above the temperature at which H_2_BDC is desorbed from the pores. The area of the rhombic pores was also calculated, using the distances between the four Al ions surrounding this opening (Supplementary Fig. 12e) which are uniquely determined by the unit cell parameters. Importantly, the high temperature cell parameters (and hence pore opening area) are broadly unchanged upon cooling the sample to room temperature, and confirm that the MIL-53-lp phase pores do not close upon cooling and atmospheric water sorption. Meanwhile, during the thermal treatment process the glass-forming phase (ZIF-62) in (MIL-53/ZIF-62)(25/75) behaves similarly to that of pure ZIF-62 (Supplementary Fig. 12f, Supplementary Table 1).

Electron microscopy was used to investigate the crystal–glass microstructure in both (MIL-53)_0.25_(*a*_g_ZIF-62)_0.75_ and (UiO-66)_0.25_(*a*_g_ZIF-62)_0.75_ CGCs. STEM electron energy loss spectroscopy (STEM-EELS) measurements demonstrated characteristic signatures corroborating the co-location of carboxylate and imidazolate ligands in the respective MIL-53 (or UiO-66) and ZIF-62 glass domains (Supplementary Figs. 13 and 14)^34^. STEM-EDS was also used to map the elemental distribution of the metal centres, demonstrating a mixture of the two separated phases in the (MIL-53)_0.25_(*a*_g_ZIF-62)_0.75_ CGC. Experiments on a (UiO-66)_0.25_(*a*_g_ZIF-62)_0.75_ CGC also revealed two separated phases at the nanoscale level (Supplementary Figs. 15 and 16).

Two-dimensional STEM-EDS mapping indicated near-homogeneous mixing of the two phases, though the distribution in three-dimensional space remained unknown. STEM-EDS tomography was used to reconstruct a complete shard of (MIL-53)_0.25_(*a*_g_ZIF-62)_0.75_ CGC (Fig. 2b and Supplementary Fig. 17). This revealed MIL-53 particles of between 30 and 300 nm in size, evenly embedded within the ZIF-62 glass substrate. The degree of surface-facing MIL-53 phase in the reconstructed particles may indicate increased preference for fracturing at MIL-53/ZIF-62 interfaces (Supplementary Fig. 17). Similar results were also obtained with the (UiO-66)_0.25_(*a*_g_ZIF-62)_0.75_ CGC (Supplementary Fig. 18).

SED has recently emerged as an effective way to obtain nanoscale structural insight from beam sensitive materials^35^. Here, the number of Bragg diffraction discs found in the diffraction pattern recorded at each probe position was plotted to reveal the location of crystalline phases, as shown in Fig. 2c and Supplementary Fig. 19. These crystallinity maps demonstrated close contact between crystalline and non-crystalline regions within the composites. Comparison with compositional maps obtained via STEM-EDS mapping of the same particles, showing the distribution of the metal centres, confirm that the crystalline regions correspond to the metal-centres expected for the MIL-53 and UiO-66 crystals in each CGC material.

**Fig. 3:**
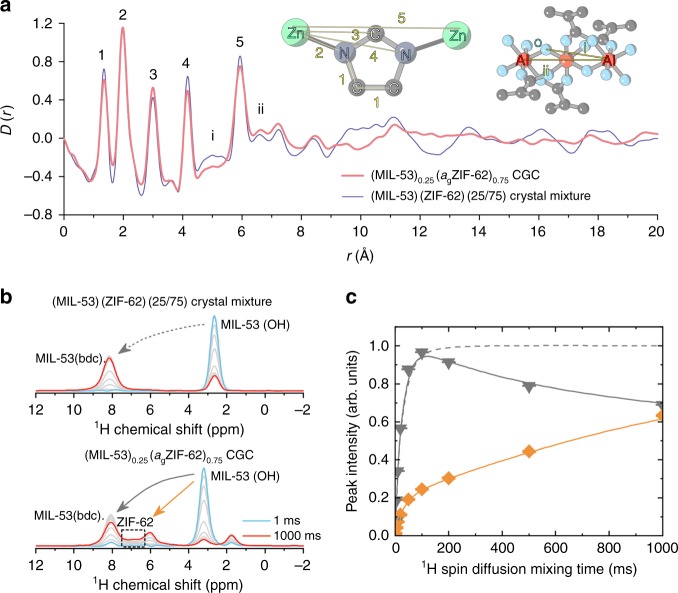
Phase distribution in the (MIL-53)(ZIF-62)(25/75) and (MIL-53)_0.25_(*a*_g_ZIF-62)_0.75_ CGC. **a** Pair distribution function (PDF) *D(r)* calculated via Fourier transform of the X-ray total scattering structure factor *S(Q)* for the crystal mixtures and CGC. The inset shows the scheme for PDF peak assignment. **b** Slices along the indirectly detected dimension through the 2D ^1^H spin-diffusion NMR spectra. Slices were taken at chemical shift of about 3 ppm, and show how polarisation transfer between the OH groups of MIL-53 and all other protons of the samples proceeds. **c** Spin-diffusion curves obtained by extracting integrated intensities of selected peaks within the slices through the 2D ^1^H spin-diffusion NMR spectra. Dashed grey line describes proton polarisation transfer between OH groups and BDC linkers of the crystal mixture, grey triangles and solid line describe polarisation transfer between OH groups and BDC linkers of MIL-53 within CGC, and orange diamond signs and solid line describe polarisation transfer between the OH groups of MIL-53 and imidazolate linkers of ZIF-62 within CGC

Synchrotron X-ray total scattering measurements were also performed *ex-situ* on both crystalline mixtures and CGC samples (Supplementary Fig. 20). Both crystalline mixtures studied contained extensive Bragg diffraction in their structure factors, which reduced upon crystal-glass formation. The X-ray PDFs, *D(r)*, were extracted after appropriate data corrections (Fig. 3a and Supplementary Fig. 21)^36^. The PDFs of (MIL-53)(ZIF-62)(25/75) and (MIL-53)_0.25_(*a*_g_ZIF-62)_0.75_ CGC were very similar below 7.5 Å, i.e., in their short range order. The similarity in short-range Al and Zn correlations, between both crystalline mixtures and CGC confirms the structural integrity of each component. As expected, the PDFs of the composite retain the longer-range oscillations due to Al-Al correlations in MIL-53 exceeding 8 Å. However, the majority of the long-range interatomic correlations broaden and weaken after melting and vitrification of ZIF-62, which is the dominant-scattering component of the CGC. The contribution to the X-ray scattering from UiO-66 is greater than that of MIL-53 in their respective CGCs with ZIF-62 because of the heavier Zr atoms in UiO-66 and hence changes to the correlations from UiO-66 are more clearly seen in the PDF. We observe that the PDF of (UiO-66)_0.25_(*a*_g_ZIF-62)_0.75_ CGC contains weaker features above 6.4 Å compared to the PDF from (UiO-66)(ZIF-62)(25/75), which is broadly consistent with a partial structural degradation of the UiO-66 component.

To confirm the STEM observationsof a homogeneous distribution of MIL-53 particles within the *a*_g_ZIF-62 matrix, ^1^H and ^13^C magic angle spinning (MAS) solid state NMR spectroscopic measurements were performed on the (MIL-53)(ZIF-62)(25/75) crystal mixture and (MIL-53)_0.25_(*a*_g_ZIF-62)_0.75_ CGC (Supplementary Fig. 22). Peaks assigned to the bIm and Im linkers in ZIF-62, as well as the peaks of the BDC linker in MIL-53, are broadly similar for both samples. Peaks belonging to the hydrated form of MIL-53 (MIL-53-np) were not observed in the NMR spectrum of (MIL-53)_0.25_(*a*_g_ZIF-62)_0.75_ CGC, suggesting only the dehydrated open pore form of MIL-53 (MIL-53-lp) is present within the MOF CGC^28,37^.

In previous work^38^, we have used spin-diffusion NMR spectroscopy in order to investigate the distribution of organic components within mixed-linker MOF systems. This technique makes use of the differential rates of proton polarisation transfer between species on the same, or separate, organic linkers (Supplementary Fig. 23). Analysis of two series of spin-diffusion NMR spectra of (MIL-53)(ZIF-62)(25/75) crystal mixture and (MIL-53)_0.25_(*a*_g_ZIF-62)_0.75_ CGC (Fig. 3b, c) shows a significant difference between the two samples (Fig. 3b). Within the figure, blue lines represent the slices through the spectra measured at spin-diffusion mixing time of 1 ms, red lines correspond to mixing time of 1000 ms, and grey lines correspond to mixing times of 2, 5, 10, 20, 50, 100, 200 and 500 ms. In both samples, a peak at about 8 ppm is due to protons on BDC linkers, and a peak at close to 3 ppm is due to protons of the bridging OH groups of the inorganic chains of MIL-53. The proton peak of ZIF-62 appears at 6.8 ppm. Whereas in the crystal mixture no proton polarisation transfer between MIL-53 and ZIF-62 is detected, transfer of polarisation between the OH protons of MIL-53 and the imidazolate-based protons of *a*_g_ZIF-62 is observed in the CGC. The fast polarisation transfer (steep curve) for short mixing times is indicative of the close contacts between MIL-53 and ZIF-62 domains in the CGC (Fig. 3c). The fact that the curve shown does not reach a plateau, and is in fact still rising at mixing times as long as 1 s, suggests that the OH groups in MIL-53 crystals and imidazolate linkers in *a*_g_ZIF-62, are present within distinct domains. If they were present within the same framework, the polarisation–transfer curve would resemble the one that describes transfer between the OH groups and BDC linkers of MIL-53, which reaches a plateau at about 200 ms (Fig. 3c).

Results of NMR measurements were less informative for the UiO-66 derived samples. ^1^H and ^13^C MAS NMR spectra detected some changes induced by melting (Supplementary Fig. 24): in the proton spectrum the OH peak at 0.3 ppm disappeared, and in both the proton and the carbon spectra, the BDC peaks became considerably broader, confirming the partial degradation of UiO-66 in contact with the ZIF-62 liquid. ^13^C-detected proton-spin-diffusion NMR experiments on both UiO-66 derived samples did not indicate close proximity of BDC and imidazolate linkers.

### Density and mechanical properties

One benefit of the CGC is their processability, which will enable the material to be shaped for different applications. The density and mechanical properties can provide important information on whether these materials will withstand industrial conditions^11^. The densities of the crystalline mixtures, and of the CGC samples, were measured with gas pycnometry. The densities of the CGCs were all higher than the corresponding initial crystal powder mixtures, e.g., from 1.62 ± 0.03 to 1.78 ± 0.08 g/cm^3^ for (MIL-53)(ZIF-62)(25/75) crystal and (MIL-53)_0.25_(*a*_g_ZIF-62)_0.75_ CGC, respectively (Supplementary Fig. 25). The mechanical properties of the composite glass samples were probed by nanoindentation on polished surfaces (Supplementary Figs. 26 and 27). The Young’s modulus (*E*) increased for both (MIL-53)_0.25_(*a*_g_ZIF-62)_0.75_ (*E* ≈ 7.7 GPa) and (UiO-66)_0.25_(*a*_g_ZIF-62)_0.75_ (*E* ≈ 7.9 GPa) compared with the pure *a*_g_ZIF-62 counterpart (*E* ≈ 5.8 GPa), which correlates well with their densities, as well as the larger (obtained through quantum chemistry calculations) elastic moduli of both MIL-53 (*E* = 25 GPa) and UiO-66(Zr) (*E* = 49 GPa)^39,40^. This observation suggests the composite glass has improved mechanical rigidity against irreversible plastic deformation.

### Stabilisation of open pore MIL-53 and CGC porosity

**Fig. 4:**
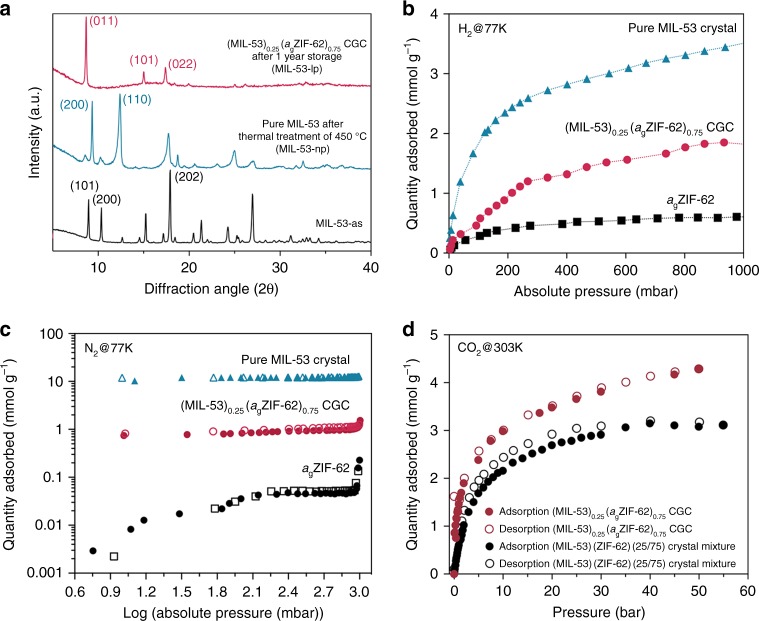
Stabilisation of MIL-53-lp in CGC and gas adsorption. **a** XRD pattern of the MIL-53-as, MIL-53 crystal after thermal treatment (MIL-53-np) and CGC. All measurements were conducted at ambient conditions. **b** H_2_ adsorption profiles at 77 K. **c** N_2_ adsorption(solid)/desorption(open) profiles at 77 K. **d** High pressure CO_2_ adsorption (solid)/desorption (open) isotherms of the crystalline mixture (black) and CGCs (red) performed at 303 K

The close interaction observed between MIL-53 crystallites and the ZIF-62 glass matrix in (MIL-53)_0.25_(*a*_g_ZIF-62)_0.75_ CGC led us to probe the effect of encapsulation upon stabilisation of MIL-53-lp structure. The XRD pattern of (MIL-53)_0.25_(*a*_g_ZIF-62)_0.75_ CGC indicated that only the open-pore MIL-53-lp was present, i.e., that rehydration and transition to close-pore MIL-53-np, does not occur, even after 1-year storage at ambient conditions (Fig. 4a). This confirms that the formation of the open pore structure of MIL-53 within the CGC is not accompanied by hysteresis^33^. This is not the case for pure samples of MIL-53(Al)-lp, which reversibly adsorb water molecules at room temperature and undergo pore shrinkage to the closed pore phase (MIL-53-np) within an hour^28^.

The fact that the MIL-53-lp to MIL-53-np transition does not occur, even when exposed to air at ambient conditions, provides a distinct advantage for the use of MOF CGCs. The effect was previously predicted, both from analytical mechanics and numerical finite-element methods^41,42^, but has not, thus far, been experimentally observed. If a composite is formed that includes a soft porous framework, such as the breathing MIL-53, and a compatible polymeric phase featuring normal mechanical properties with no possibility for guest-induced structural transitions (here represented by *a*_g_ZIF-62), the resulting behaviour of the composite will be dominated by the polymeric phase and the capacity for breathing of the MIL-53 crystals embedded in it will be lost.

A range of gas adsorption isotherm experiments were performed on the MIL-53 CGC samples to determine the effect of encapsulation on gas adsorption behaviours. *a*_g_ZIF-62 has been previously demonstrated to possess accessible, permanent porosity toward both H_2_ and CO_2_^43^, with uptake capacities of 0.40 mmol H_2_/g at 77 K and 0.90 mmol CO_2_/g at 273 K, respectively. Measurements were repeated here, and, as expected, the ZIF-62 glass is porous to small gas molecules (with H_2_ of 2.9 Å kinetic diameter uptake of 0.62 mmol/g at 1 bar and 77 K, Fig. 4b). The incorporation of crystalline MIL-53 improves the H_2_ uptake of *a*_g_ZIF-62 to *ca*. 1.9 mmol/g at 1 bar. This can be attributed to the high measured gas adsorption capacity of pure MIL-53 (open pore structure) at 3.55 mmol/g, which aligns with the reported values^44,45^. In comparison, N_2_ adsorption isotherms at 77 K of the *a*_g_ZIF-62 and the MOF–crystal composite glass show very little adsorption relative to that of crystalline MIL-53, as the analyte gas appears too large (3.6 Å kinetic diameter) to penetrate through the *a*_g_ZIF-62 network (Fig. 4c). This is in accordance with previous literature on *a*_g_ZIF-62^43^. Ar (3.4 Å kinetic diameter) adsorption experiments were carried out at 87 K, (Supplementary Fig. 28), and demonstrate similar inaccessibility of the glass component to large analyte molecules. Though the low adsorption severely limits precision, pore size distributions gained from Ar isotherms on (MIL-53)(ZIF-62)(25/75) and (MIL-53)_0.25_(*a*_g_ZIF-62)_0.75_ CGC demonstrate pores at 5–6 Å for both crystalline mixture and CGC (Supplementary Fig. 29), in addition to one at ca 11 Å for the CGC.

Using an analyte gas with a slightly smaller critical diameter, CO_2_ (3.3 Å kinetic diameter), we find that the gas adsorption capacity of (MIL-53)_0.25_(*a*_g_ZIF-62)_0.75_ CGC approaches the capacity of a sample of pure MIL-53 at the same condition (273 K, 1 bar, Supplementary Fig. 30). High-pressure CO_2_ adsorption experiments were performed on both (MIL-53)(ZIF-62)(25/75) and (MIL-53)_0.25_(*a*_g_ZIF-62)_0.75_. The CGC demonstrated an improvement of ca. 30% in CO_2_ adsorptive capacity at 303 K and 50 bar (Fig. 4d). This phenomenon can be explained by considering the breathing behaviour of MIL-53. Below pressures of 3 bar, the adsorption of CO_2_ results in weak dipolar or quadrupolar host–guest interactions. This interaction causes the unit cell to contract to a narrow pore structure with a relatively low adsorption capacity of *ca*. 2.5 mmol/g^46^. At higher pressures above 10 bar, the pores of the framework are re-opened, increasing the CO_2_ adsorption capacity to 8–9 mmol/g^47^. In comparison, the stabilisation of open-pore MIL-53 within the composite glass readily allows a high CO_2_ adsorption quantity even at low pressure conditions (up to 1 bar)—although the narrow pore phase has higher affinity for CO_2_, as can be seen in the very low pressure region (<100 m bar). Based on the composition of the CGC, the estimated CO_2_ adsorption is 2.71 mmol/g, which is lower than the experimental results. This suggests that the excess CO_2_ uptake observed here may be partially ascribed to a small amount of mesopores within CGC, arising from the interface between crystal and glass components (Supplementary Fig. 31). CO_2_ isotherms measured on the CGC samples at 273 K were accompanied with hysteresis for the desorption cycles, which has also been observed previously in pure *a*_g_ZIF-62 samples^43^.

Water adsorption experiments were also performed on both (MIL-53)(ZIF-62)(25/75) and (MIL-53)_0.25_(*a*_g_ZIF-62)_0.75_ CGC (Supplementary Fig. 32). An abrupt uptake at 60% relative humidity is noted for both samples during the first cycle, whilst subsequent cycles showed a better cyclability and higher amount adsorbed for the (MIL-53)_0.25_(*a*_g_ZIF-62)_0.75._ The uptake of H_2_O here implies that the stabilisation of MIL-53-lp does not arise because it is excluded from entering the composite CGC material. Instead, we suggest that the polymeric phase is not soft enough to accommodate a large-scale change of the crystal phase structure, while the interfacial contact between the two phases is maintained.

## Discussion

Composite formation has been used to exert control over the chemical functionality and physical properties of materials such as molecular crystals^48^. Here, this approach has been adapted to metal–organic frameworks. We believe this to be a prototypical example of a MOF-CGC, here formed by embedding a MIL-53 within a MOF–glass matrix. The structural integrity of both the crystalline and glass components of the materials has been demonstrated for samples both before and after vitrification. In this material, two separated phases are in close proximity and well mixed at a nanoscale. The glass matrix stabilises the phase transition of flexible MIL-53, maintaining its open pore structure at ambient conditions, which leads to significant improvement of gas adsorption at room temperature. We, therefore, hypothesise that a glass matrix support may hinder temperature-dependent structural rearrangements in other MOFs. In addition, this family of composite materials may also facilitate the assembly of discrete MOF crystal particles into thermally and mechanically stable devices with various shapes, such as adsorption column or molecular separation membranes.

## Methods

### Synthesis

ZIF-62: Zinc nitrate hexahydrate (1.65 g, 5.54 × 10^−3^ mol) and imidazole (8.91 g, 0.13 mol) were added to a 200 mL screw top jar, dissolved in N,N-dimethylformamide (DMF, 75 mL) and stirred for 1 h. Once complete dissolution was achieved, benzimidazole (1.55 g, 1.31 × 10^−2^ mol) was added and heated to 130 °C for 48 h. The product was allowed to cool to room temperature and crystals were separated by vacuum assisted filtration and washed with DMF (60 mL) and dichloromethane (DCM) (40 mL) before being dried in a vacuum oven at 150 °C for 24 h^23^.

MIL-53: Aluminium nitrate nonahydrate (26.00 g, 6.93 × 10^−2^ mol) and terephthalic acid (5.76 g, 4.96 × 10^−2^ mol) were dissolved in water (100 ml) and placed into a Teflon-lined autoclave and placed in an oven at 220 °C for 72 h. The resulting powder was washed with deionised water (3 × 30 ml) and dried in a vacuum oven at 150 °C for 24 h^28^.

UiO-66: Zirconium(IV) chloride (0.59 g, 2.53 × 10^−3^ mol) and terephthalic acid (0.63 g, 3.79 × 10^−3^ mol) were dissolved in DMF (75 ml) with hydrochloric acid (37 wt%, 0.37 ml) and glacial acetic acid (99.99%, 0.75 ml) and placed into a Teflon-lined autoclave. The mixture was then placed in an oven at 120 °C for 96 h. The product was allowed to cool to room temperature and crystals were separated by vacuum assisted filtration and washed with DMF (60 mL) and DCM (40 mL) before being dried in a vacuum oven at 150 °C for 24 h^30^.

### Fabrication of composite crystal–glass

A series of CGCs with different concentrations of MIL-53 or UiO-66 were prepared. Different compositions were made and the resultant mixtures are referred to as (Crystal)(Glass–former)(X/Y), where *X* and *Y* are percentage by mass of each component. For example, a 25 wt% sample of crystalline MIL-53 and 75 wt% crystalline ZIF-62 sample is referred to as (MIL-53)(ZIF-62)(25/75).

The separate crystalline components were mixed by ball milling at 30 Hz for 5 min with a 7 mm stainless steel ball. The short milling time was applied to avoid crystal amorphisation caused by the mechanical stress. Subsequently, the crystal mixtures were placed in a tube furnace for thermal treatment with a ramping rate of 10 °C min^−1^ under argon (Ar) protection. The sample was held at 450 °C for 10 min and then cooled back to room temperature under Ar protection. The CGCs are referred to as (Crystal)_X_(*a*_g_Glass–matrix)_Y_, in line with our previous publication^32^. Pure phase crystals were also subjected to the same ball milling and thermal treatment as a benchmark^32^.

### Powder XRD analysis

Room temperature powder XRD analysis (2*θ* = 5°–40°) were collected with a Bruker-AXS D8 diffractometer using Cu K_α_ (*λ* = 1.540598 Å) radiation and a LynxEye position-sensitive detector in Bragg–Brentano parafocusing geometry. The 2*θ* step size was 0.02°, with 10 s per step.

### Thermogravimetric and calorimetric analysis

Thermogravimetric analysis (TGA) and DSC analysis were conducted using a TA instrument STD Q600. The MOF powder samples were placed in a ceramic crucible situated on a sample holder, and then heated at 10 °C min^−1^ to above the melting temperature of ZIF-62 under an Ar environment. For the two-cycles of TGA/DSC upscan, after the first upscan the sample was cooled back to 30 °C at 10 °C min^−1^ under an Ar environment, and then ramped up to the targeted temperature at the rate of 10 °C min^−1^ for the second upscan.

### Scanning electron microscopy

The surface morphologies of the crystal mixture and composite glass samples were investigated using a high-resolution scanning electron microscope, FEI Nova Nano SEM 450, under the backscattering mode. All samples were dried at 30 °C followed by chromium coating prior to imaging.

### In situ synchrotron powder diffraction

In situ synchrotron data were collected at the SAXS beamline of the Australian Synchrotron facility. Dried crystal powder samples were loaded into 1.0 mm quartz capillaries under Ar protection in a glove box. The in situ synchrotron powder diffraction was investigated with SAXS beamline at 16 keV, 2675 mm camera length using a Pilatus 1 M detector in transmission mode. For each analysis, a line scan of 3 mm at 0.3 mm s^−1^ was conducted. The background of the empty capillary was subtracted. The data were processed using in-house developed Scatterbrain software for averaging and background subtraction.

### Scanning transmission electron microscopy

STEM EDS, EELS and tomography were performed using an FEI Osiris microscope (Thermo Fisher Scientific) equipped with a high-brightness X-FEG electron source and operated at 80 kV. The beam convergence semi-angle was set to 11.0 mrad. For EELS, the collection semi-angle was estimated as 40.8 mrad. EDS was acquired using a Super-X EDS detector system with four detectors mounted symmetrically about the optic axis of the microscope. For STEM-EDS tomography, EDS spectrum images were  acquired over a tilt-series from −65° to 75° in 10° increments using a Fischione tomography holder, with a probe current estimated at <450 pA. Pixel dwell times were increased at high tilt due to the large number of copper counts at increasing tilt-angle.

Data were processed using Hyperspy^49^, an open-source software coded in Python. Maps of each X-ray emission line of interest (Zr *L*_*α*_, Al *K*_*α*_, Zn *K*_*α*_) were first extracted by integrating in an energy window, background subtracted by linear interpolation from adjacent regions of the spectrum without other X-ray peaks present. Map intensities were then re-normalised such that the total intensity of each element was constant throughout the tilt-series, a valid assumption for particles where the mass of the element in the field of view is constant throughout the tilt-series. The re-normalised maps were then aligned by centre-of-mass, and the tilt-axis was adjusted using Scikit-Image, an open source image processing software coded in Python, by applying shifts and rotations to minimise artefacts in back-projection reconstructions. A compressed sensing reconstruction algorithm coded in MATLAB (Mathworks) was used to perform independent reconstructions of the metal-centre spatial distributions. This compressed sensing tomography implementation used three-dimensional total generalised variation^50^ regularisation in conjunction with a real-space projector from the Astra toolbox^51^ and using the primal-dual hybrid gradient method^52^ to solve the reconstruction problem. Reconstructions were visualised in Avizo (Thermo Fisher Scientific) software without any further image processing. Visualisations are presented as volume renderings where each volume element is assigned a colour and relative solid appearance based on the intensity at the corresponding volume elements of the reconstruction. Visualisations for each independent element reconstruction were superimposed in the final visualisations and a selection of cuts through the volume were used to examine sub-surface features.

SED involves the acquisition of a two-dimensional electron diffraction pattern at every position as a narrow electron probe is scanned across the specimen. When the electron probe is positioned on a crystalline region of material, strong diffraction to Bragg reflections will typically be observed, whereas when the electron probe is positioned on non-crystalline material no sharp Bragg reflections will be measured. Determining probe positions at which sharp diffraction peaks are recorded therefore provides a way to directly observe crystalline and non-crystalline regions. This was achieved by finding diffraction peaks in every measured diffraction pattern using a difference of Gaussians method, which involves subtracting a blurred version of the diffraction pattern from a less blurred version of the diffraction pattern, as implemented in the pyXem library.

SED was performed using a JEOL ARM300F at the Diamond Light Source, UK fitted with a high-resolution pole piece, cold field emitter, and JEOL spherical aberration correctors in both the probe forming and image forming optics. The instrument was operated at 200 kV and aligned in a nanobeam configuration using the corrector transfer lenses and a 10 µm condenser aperture to obtain a convergence semi-angle of ~1.6 mrad and a diffraction limited probe diameter ~1.6 nm. Data were acquired with a scan step size of ~4 nm and a camera length of 15 cm. The probe current was ~14 pA. A Merlin-medipix direct electron detector^53,54^, which is a counting type detector, was used to record the electron diffraction pattern at each probe position with an exposure time of 0.5 ms per probe position leading to a total electron fluence of ~200 eÅ^−1^ based on the probe current, exposure time, and assuming a disk-like probe of the diameter above. SED was acquired over a raster pattern comprising 256 × 256 probe positions and each diffraction pattern comprised 256 × 256 pixels. X-ray EDS maps were acquired from the same regions, following SED acquisition, using a larger probe current, obtained using a 150 μm condenser aperture, in order to generate sufficient X-ray counts.

### X-ray total scattering and PDF

XRD data were collected on the I15-1 beamline at the Diamond Light Source, UK using an X-ray wavelength *λ* = 0.161669 Å (76.7 keV). Crystal mixture and CGC samples were loaded into borosilicate glass capillaries of  1.5 mm (outer) diameter. Data from the samples, empty instrument and empty capillary were collected in the region of ~0.4 < *Q* < ~ 26 Å^−1^. Corrections for background, multiple scattering, container scattering, Compton scattering and absorption were performed using the GudrunX programme^55^.

### Solid-state NMR

The solid-state NMR experiments were performed on a 600 MHz Varian NMR system equipped with a 1.6 mm HXY MAS probe. All samples were spun at MAS rate of 40 kHz. Larmor frequencies for ^1^H and ^13^C were 599.47 and 150.74 MHz, respectively. The frequency axes of the recorded spectra were calibrated against the resonance frequencies of tetramethylsilane. ^1^H MAS NMR spectra were collected using Hahn-echo pulse sequence with the 90° pulse width of 1.5 μs and echo delay of a single rotation period. Sixteen scans were accumulated with the repetition delay of 5 s. ^1^H-^13^C cross-polarisation (CP) MAS NMR spectra were recorded by first exciting protons and transferring polarisation to carbon nuclei using the amplitude-ramped CP block with a duration of 4 ms. During the acquisition, a high-power two-pulse phase-modulated heteronuclear decoupling was applied^56,57^. 2D ^1^H-^1^H double-quantum single-quantum (DQ-SQ) homonuclear-correlation NMR spectrum was obtained by employing the back-to-back recoupling sequence (BABA)^58^. One BABA cycle was used for double-quantum coherence excitation and one for reconversion. A delay of 25 µs was added prior to the 90° read-out pulse of 1.65 µs. The spectral width in the indirect dimension was 40 kHz and 150 slices were accumulated along indirect dimension with 32 transients each. ^1^H-detected 2D proton spin-diffusion (PSD) spectra were measured for spin-diffusion mixing times ranging between 1 and 1000 ms. Each measurement consisted of 160 increments along t1 with 128 scans per increment and repetition delay of 0.5 s. To suppress broad peaks in the direct dimension, *T*_2_ filter was added at the end of the PSD pulse sequence. Delays before and after the 180°-pulse both lasted 2 ms. ^13^C-detected 2D PSD spectra were measured for spin-diffusion mixing times of 0 and 10 ms. Prior to the ^13^C acquisition, the polarisation was transferred between protons and carbons by utilising 1 ms CP block^59^. In this experiment, 10 increments were taken with 4096 transients each and repetition delay of 0.5 s.

### Gas pycnometry (density) and gas adsorption

Pycnometric measurements were conducted with a Micromeritics Accupyc 1340 helium pycnometer. The typical mass used for each test was around 100 mg, and the reported value was the mean and standard deviation from a cycle of 10 measurements. N_2_ and CO_2_ (at 273 K) gas adsorption isotherm measurements were conducted on a Micromeritics ASAP 2020 instrument. Around 50 mg sample was used for each measurement. All samples were degassed at 200 °C overnight prior to the adsorption/desorption test.

The argon physisorption experiments were carried out at 87 K on a BEL max apparatus (Microtrac BEL) coupled with a helium cryostat. After weighing (approx. 100 mg), the samples were outgassed to 200 °C for 10 h prior to temperature equilibration for the experiments at 87 K. A stepwise introduction of gas (argon purity 99.9999%) was employed. Helium was used for dead space calibration after the argon adsorption measurement. The micropore size distribution was calculated using the Horwath-Kawazoe method via the Saito-Foley approach.

High pressure CO_2_ adsorption at 303 K was carried out on a Rubotherm electromagnetic balance set-up (Rubotherm gmbh). After weighing (approx. 200 mg), the samples were outgassed to 200 °C for 16 h prior to temperature equilibration for the experiments at 303 K. A stepwise introduction of gas (CO_2_ purity 99.998%) was employed. Helium was used for dead space calibration prior to the CO_2_ adsorption measurements.

Water adsorption was carried out on a Hiden balance set-up (Hiden) at 298 K. After weighing (approx. 30 mg), the samples were outgassed to 200 °C for 16 h prior to equilibration at the set temperature of the experiments at 298 K. A carrier gas of nitrogen was used in which the water relative humidity was controlled between 2 and 98% in stepwise increments.

### Nanoindentation measurement

The elastic modulus (*E*) of the composite glass samples was measured using an MTS Nanoindenter XP at ambient conditions. All samples were mounted in an epoxy resin and polished using increasingly fine diamond suspension liquids. Nanoindentation experiments were conducted under the dynamic displacement controlled mode at a constant strain rate of 0.05 s^−1^. A three-sided pyramidal (Berkovich) diamond indenter tip was applied with the testing penetration depth of 500 nm. The load-displacement data collected were analysed using the Oliver and Pharr method^60^. A Poisson’s ratio of 0.4 was applied.

## Supplementary information

Supplementary Information

Peer Review File

## Data Availability

All data generated in this study are included in this Article and the Supplementary Information, and are also available from the corresponding authors upon request.

## Code availability

The code used to generate crystallinity maps (Johnstone et al., pyXem 0.7, 2019) is available at 10.5281/zenodo.2649352.
