# Supplementary material for: Metal-organic framework crystal-glass composites
Source: Nat Commun. 2019 Jun 12;10:2580. doi: 10.1038/s41467-019-10470-z (PMC6561910; doi:10.1038/s41467-019-10470-z)
Supplement: Supplementary file 1 — Supplementary Information [file 41467_2019_10470_MOESM1_ESM.pdf]

Supplementary Information for

Metal-Organic Framework Crystal-Glass Composites

Hou et al.

This PDF file includes:

Supplementary Figs. 1 to 32
Supplementary Tables 1

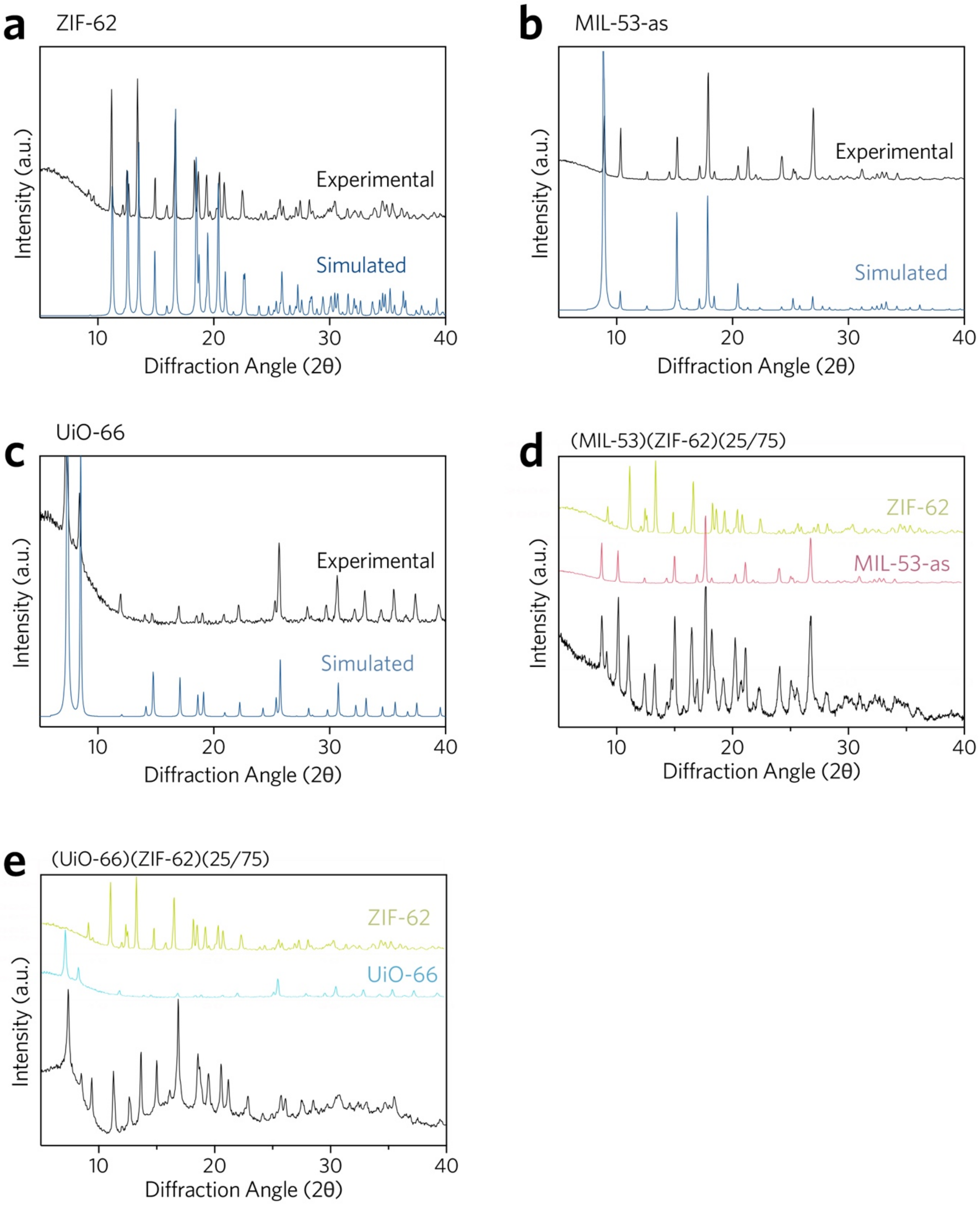

Supplementary Fig. 1. Laboratory source powder X-ray diffraction patterns of the materials. (**a**) ZIF-62, (**b**) MIL-53-as and (**c**) UiO-66, along with patterns simulated from the published cif files [1–3]**.** Powder X-ray diffraction patterns of the crystal mixtures after ball milling: (**d**) (MIL-53)(ZIF-62)(25/75), and (**e**) (UiO-66)(ZIF-62)(25/75), alongside experimental reference patterns from the pure MOFs.

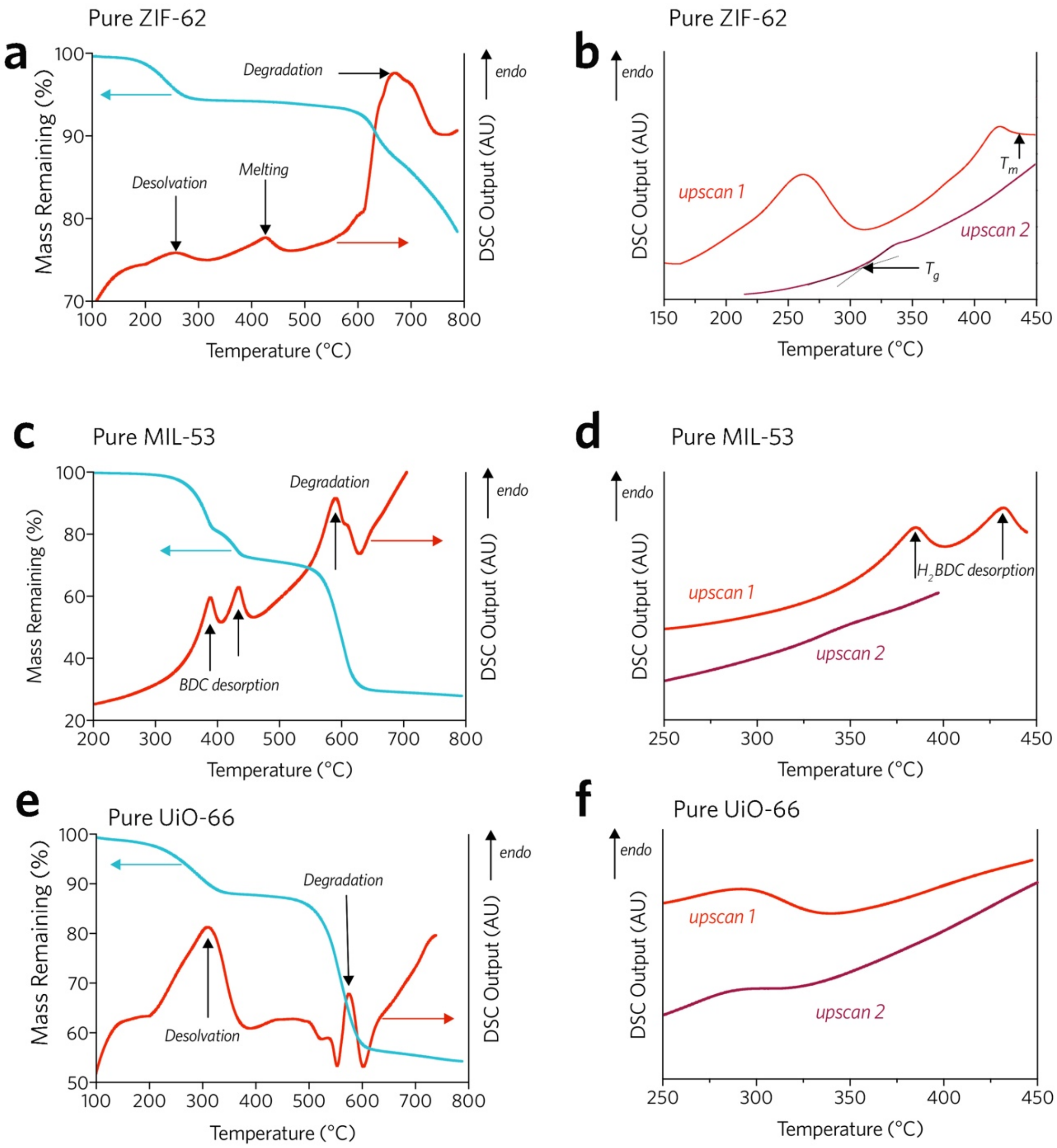

Supplementary Fig. 2. Benchmark thermogravimetric (TG) and differential scanning calorimetry (DSC) analysis for pure MOF materials. TG and DSC profile for pure ZIF-62 of (**a**) heating to 800 °C and (**b**) 2 cycles of heating to 450 °C at a rate of 10 °C/min. TG and DSC profile for pure MIL-53 upon (**c**) heating to 800 °C and on (**d**) 2 cycles of heating to 450 °C at a rate of 10 °C/min. TG and DSC profile for pure UiO-66 of heating to (**e**) 800 °C and (**f**) 2 cycles of heating to 450 °C at a rate of 10 °C/min. The thermal response at around 300 °C in the second upscan for UiO-66 can be attributed to the reversible Zr_6O_6 cluster distortion [3]. All tests were carried out under Ar environment with a flow rate of 100 mL/min.

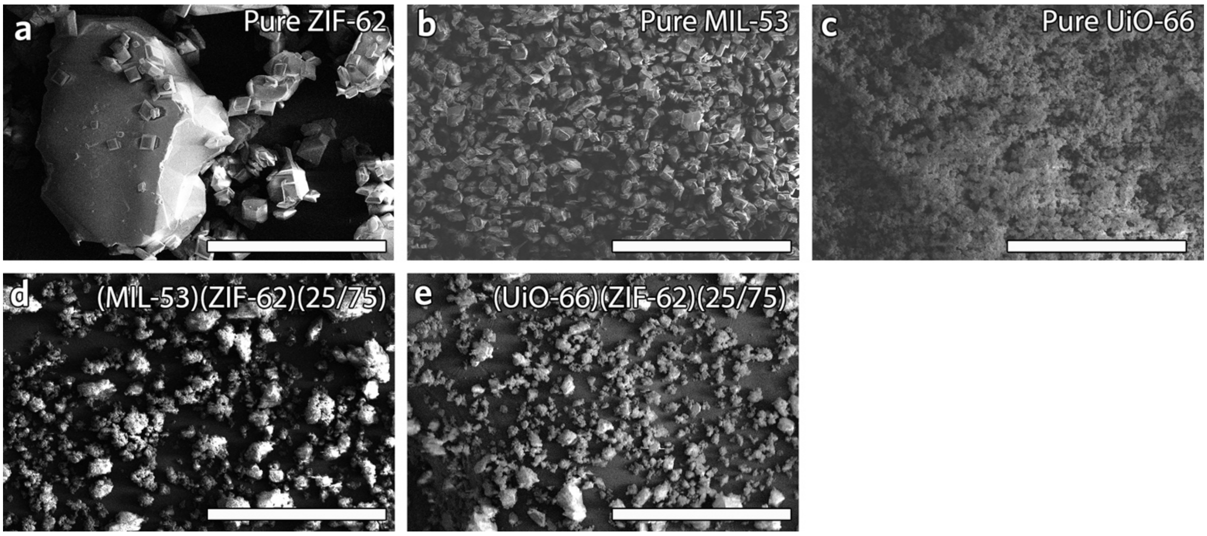

Supplementary Fig. 3. SEM images of as-synthesised and ball-milled samples. (**a**) ZIF-62, (**b**) MIL-53-as and (**c**) UiO-66. (**d**) (MIL-53)(ZIF-62)(25/75) and (**e**) (UiO-66)(ZIF-62)(25/75). Scale bars are 50 μm.

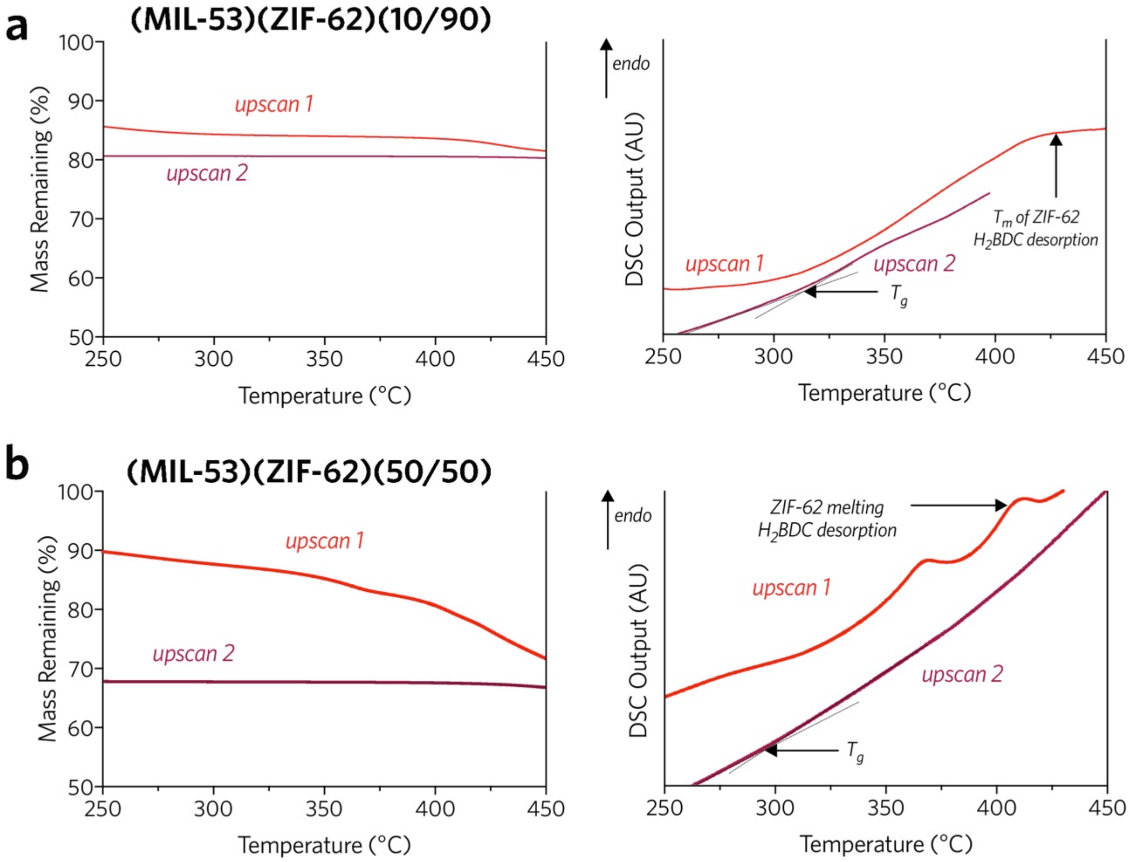

Supplementary Fig. 4. Thermogravimetric (TG, left column) and differential scanning calorimetry (DSC, right column) analysis for (MIL-53)(ZIF-62) mixtures with different MIL-53 weight percent. Mixture containing (**a**) 10 wt %, (**b**) 50 wt % of MIL-53. The heating rate of 10 °C/min, with two cycles of upscan to 450 °C.

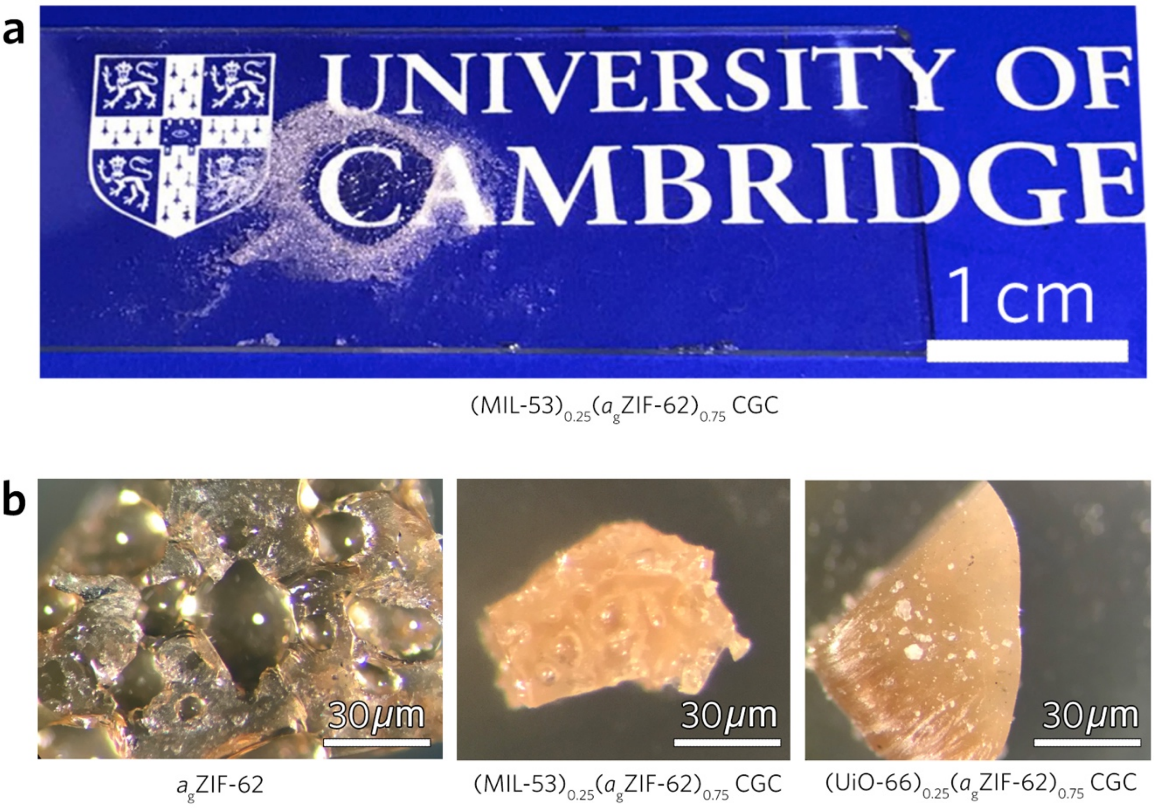

Supplementary Fig. 5. (a) Optical images of (MIL-53)$_{0.25}$(a_gZIF-62)$_{0.75}$ CGC, prepared by clamping the crystal mixture between two glass sides during heating, and (b) optical microscopic images of a_gZIF-62 and the CGCs.

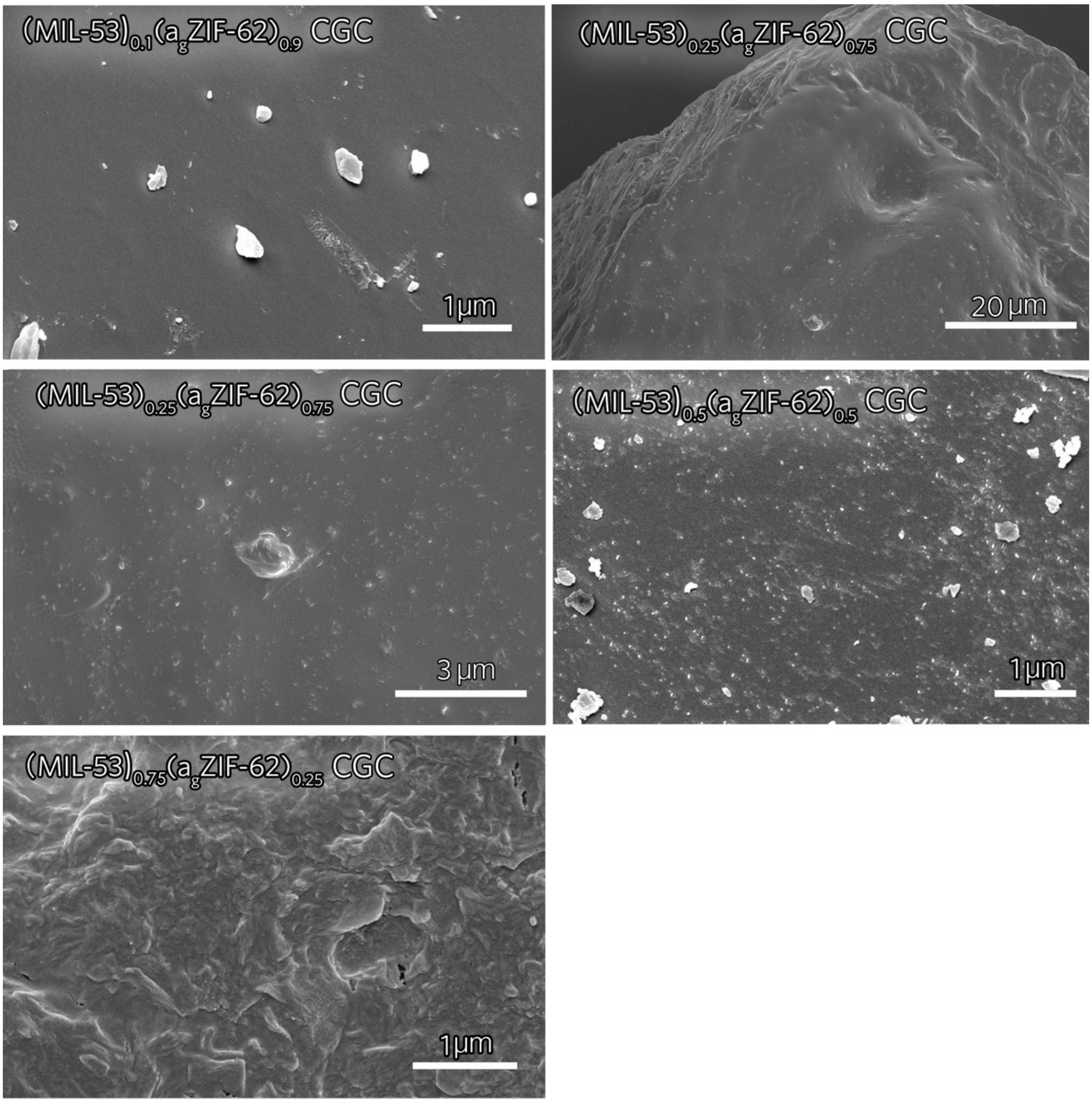

Supplementary Fig. 6. **SEM images of the CGCs with different loading of MIL-53.** All samples were held at 450 °C under Ar for 10 minutes, to ensure the complete melting of ZIF-62.

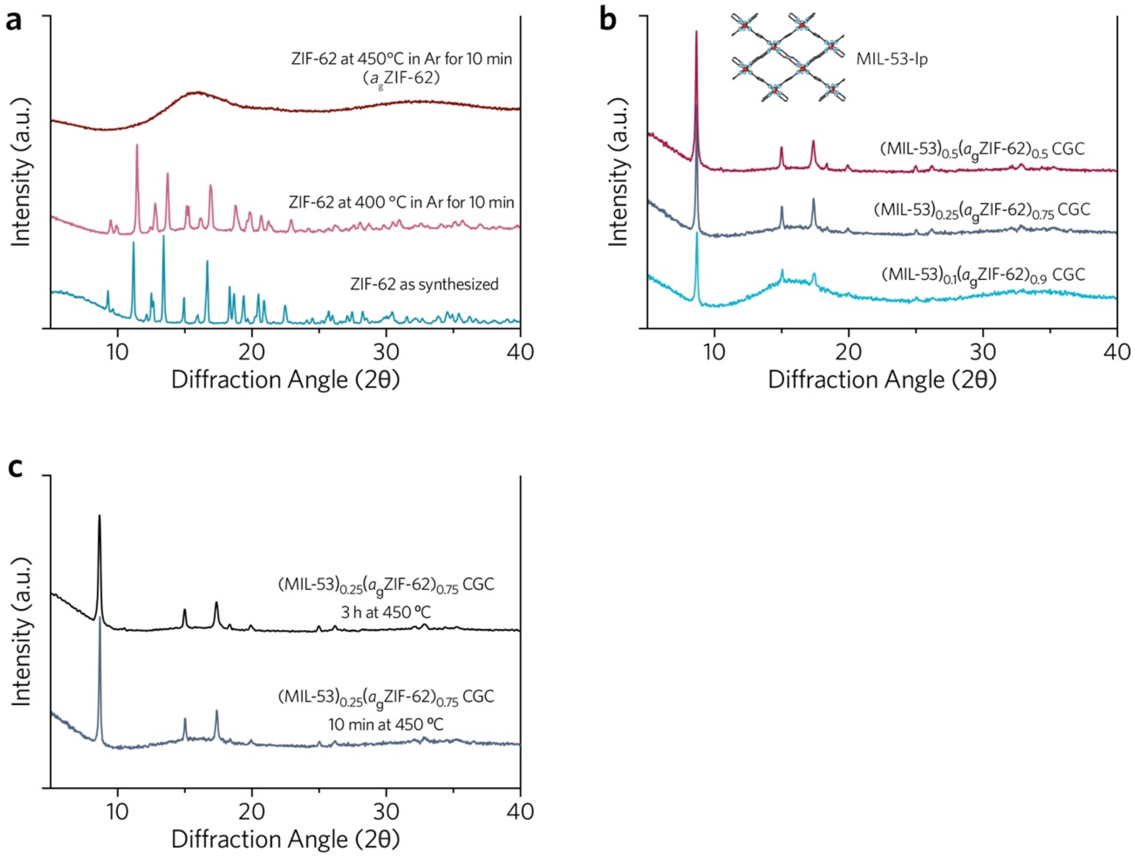

Supplementary Fig. 7. Laboratory source powder XRD pattern of the MOF glass. XRD pattern for (**a**) pure ZIF-62 after different thermal treatments and (**b**) CGC with different concentrations of MIL-53. Insert is the schematic diagram of the MIL-53-lp structure. (**c**) XRD patterns of $(\text{MIL-53})_{0.25}(a_g\text{ZIF-62})_{0.75}$ CGC with different thermal treatment time, before returning to room temperature. XRD acquisitions were conducted at ambient conditions.

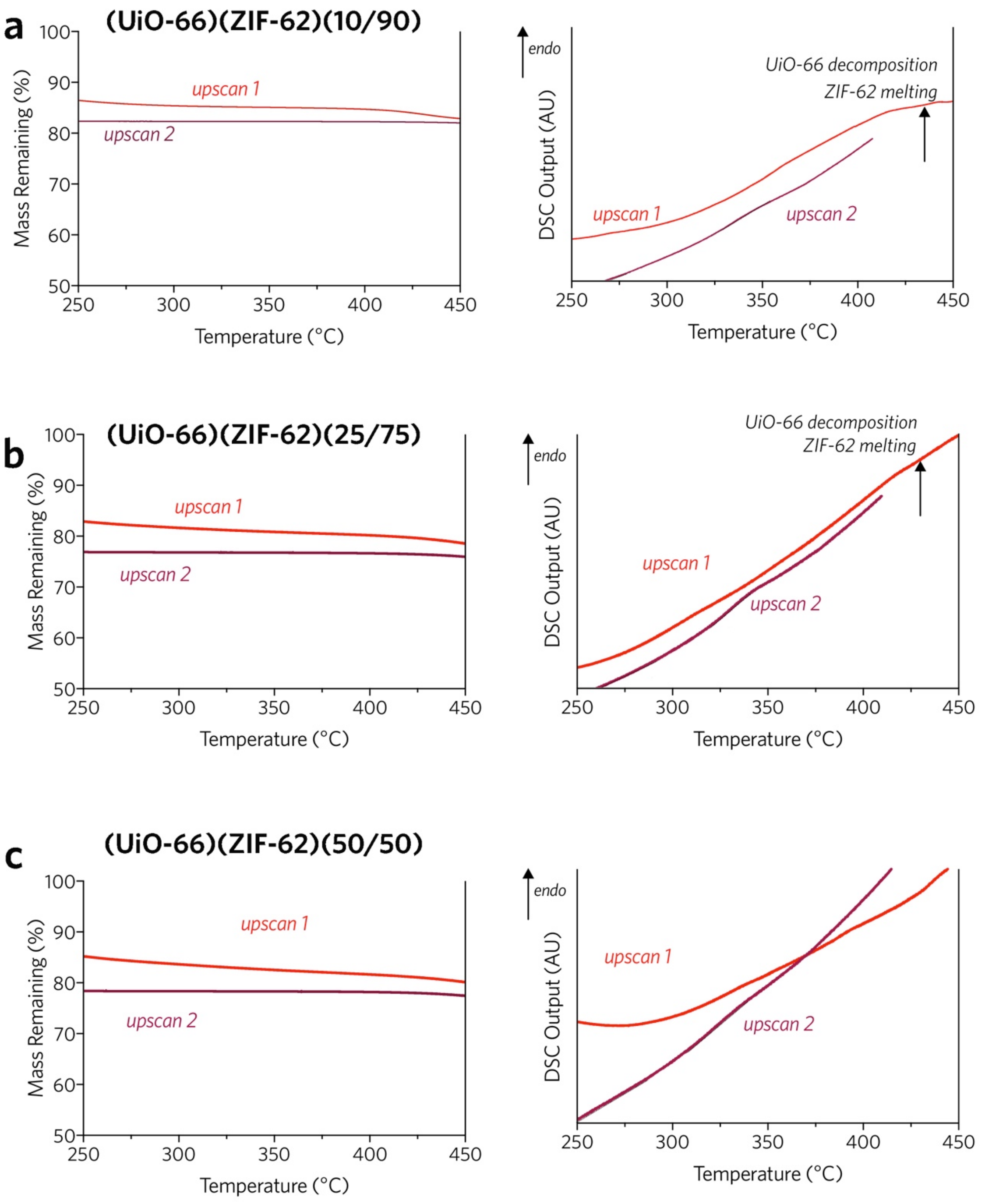

Supplementary Fig. 8. Thermogravimetric (TG, left column) and differential scanning calorimetry (DSC, right column) analysis for (UiO-66)(ZIF-62) mixtures with different UiO-66 weight percent. Mixture containing (**a**) 10 wt %, (**b**) 25 wt % and (**c**) 50 wt % of UiO-66. The heating rate of 10 °C/min, with two cycles of upscan to 450 °C.

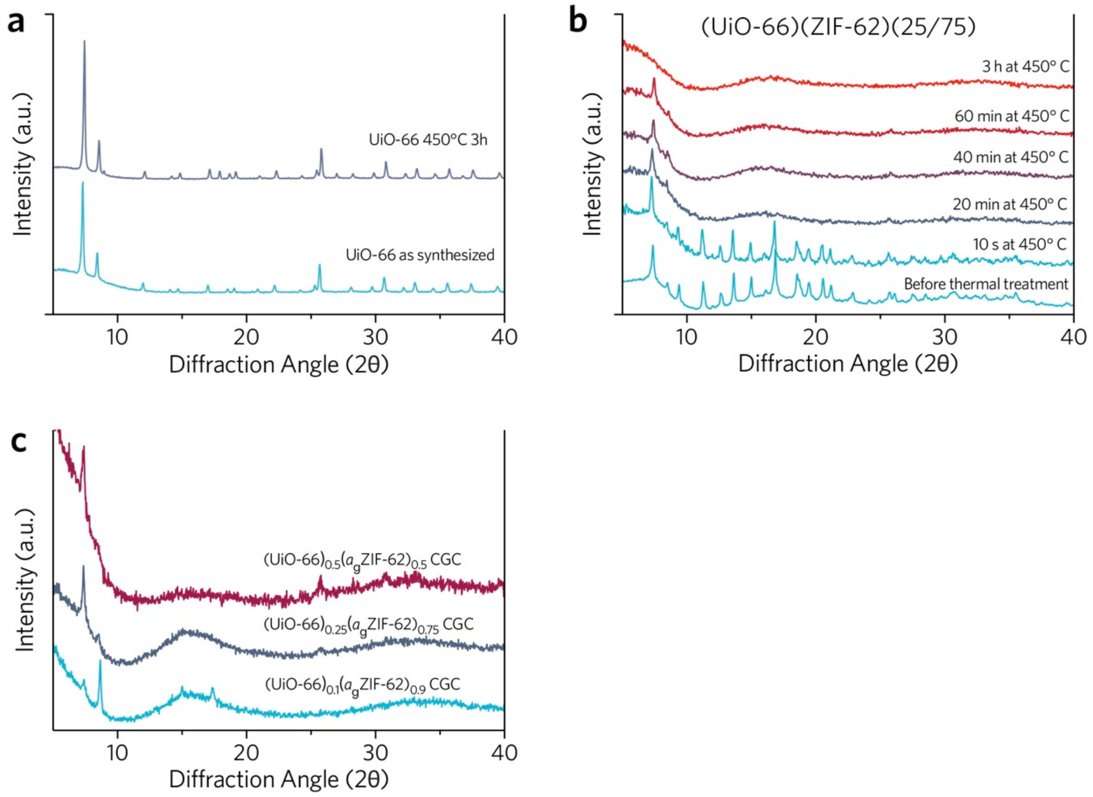

Supplementary Fig. 9. Laboratory source powder XRD patterns of the UiO-66 based CGCs. (**a**) XRD pattern of the pure UiO-66 before and after thermal treatment at 450 ºC in Ar for 3h. (**b**) Evolution of the XRD pattern of the (UiO-66)(ZIF-62)(25/75) crystal mixtures with different thermal treatment. (**c**) XRD pattern of the CGCs with different concentrations of UiO-66 (all samples were prepared with 10 min of 450 ºC thermal treatment). All powder XRD tests were conducted at ambient conditions.

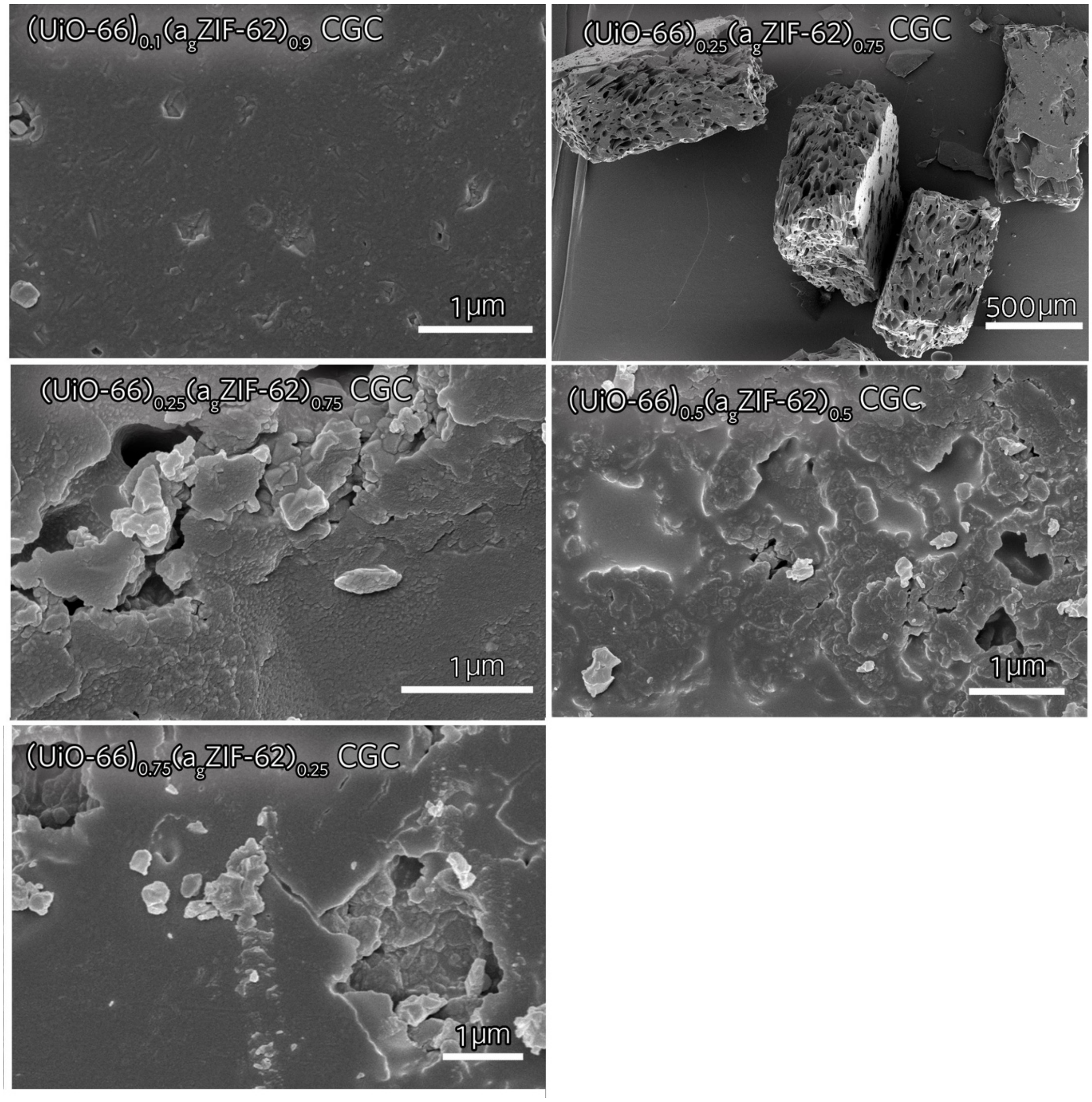

Supplementary Fig. 10. **SEM images of the CGCs with different loading of UiO-66.** All samples were held at 450 °C under Ar for 10 min to ensure the complete melting of ZIF-62.

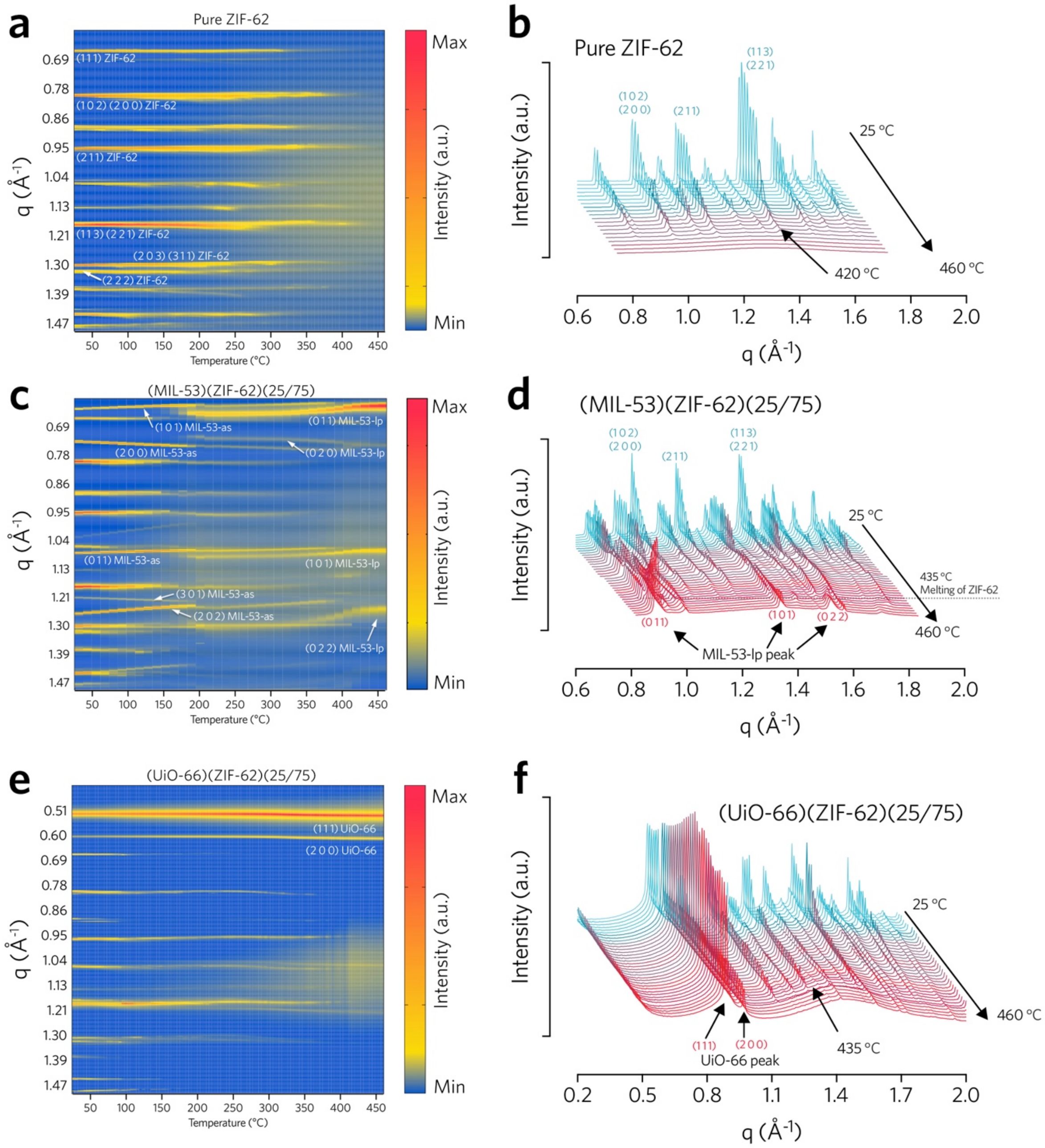

Supplementary Fig. 11. ***In-situ*** **synchrotron powder diffraction profile during the thermal treatment process (heating rate of 10 °C/min). Left column is the contour plot and right column is the waterfall plot of the same data.** (**a-b**) Pure ZIF-62, with hkl indices marked for ZIF-62. (**c-d**) (MIL-53)(ZIF-62)(25/75) crystal mixture, with hkl indices marked for ZIF-62 (blue) and MIL-53-lp (red) in the right column. (**e-f**) (UiO-66)(ZIF-62)(25/75) crystal mixture, with hkl indices marked for UiO-66 (red) in the right column.

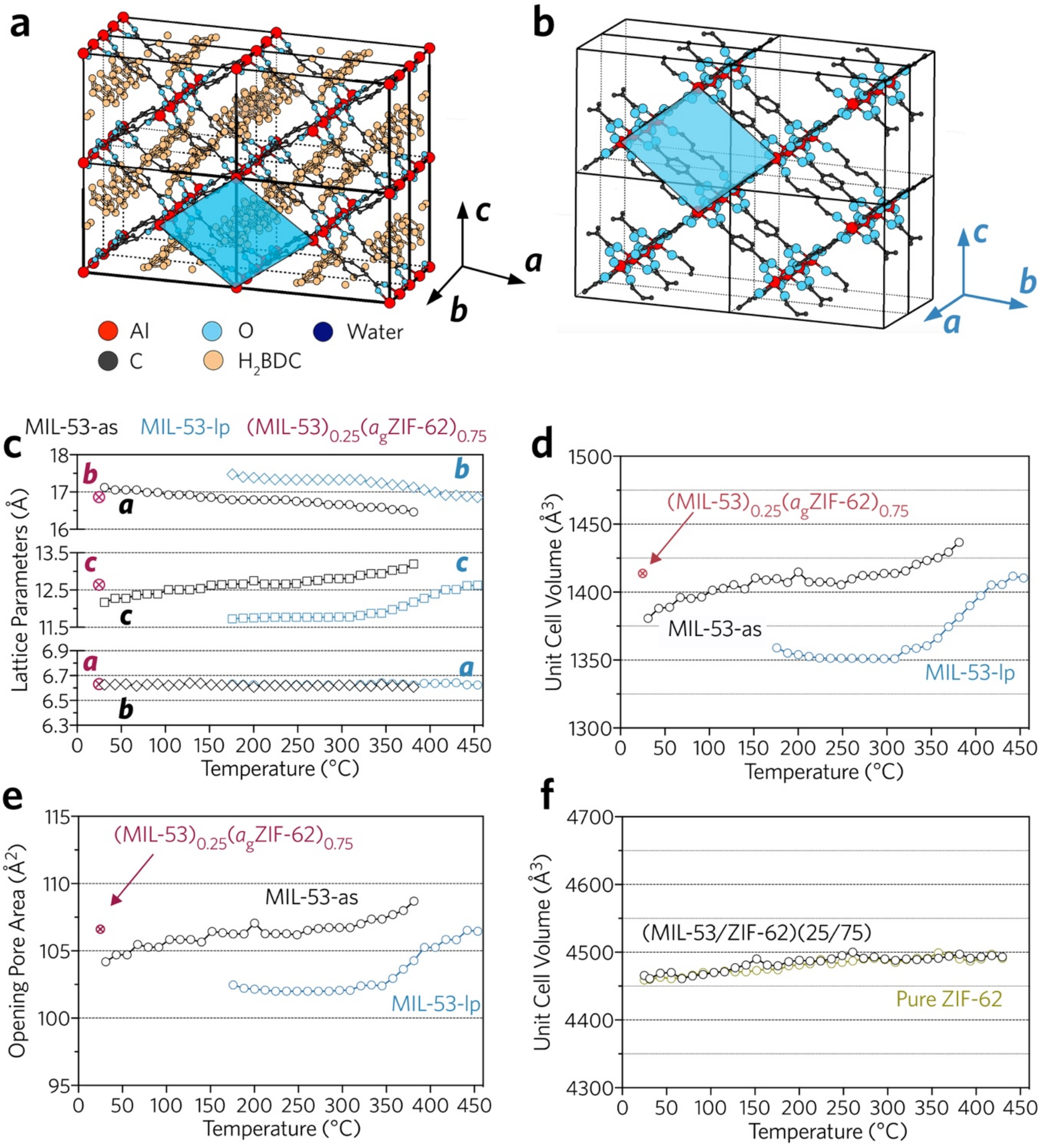

Supplementary Fig. 12. Phase transition of MIL-53 and melting of ZIF-62 during the $(MIL\text{-}53)_{0.25}(a_gZIF\text{-}62)_{0.75}$ CGC fabrication process. (**a-b**) Schematic diagram of the MIL-53-as and MIL-53-lp unit cell structure. Atom size is not to scale. (**c**) Change of the lattice parameters and (**d**) unit cell volume of MIL-53-as (black) and MIL-53-lp (blue) during the phase transition process. (**e**) Change of the opening pore area during the phase transition process. The opening pore area is indicated in the scheme a and b with blue colour. The *ex-situ* XRD patterns from samples at room temperature are highlighted in the figure (**c-e)** with red marks. (**f**) Change of the unit cell volume of ZIF-62 phase during the thermal treatment process for both pure ZIF-62 and (MIL-53/ZIF-62)(25/75). All values were fitted from the *in-situ* synchrotron powder diffraction and *ex-situ* powder XRD patterns using the published cif files [1,2].

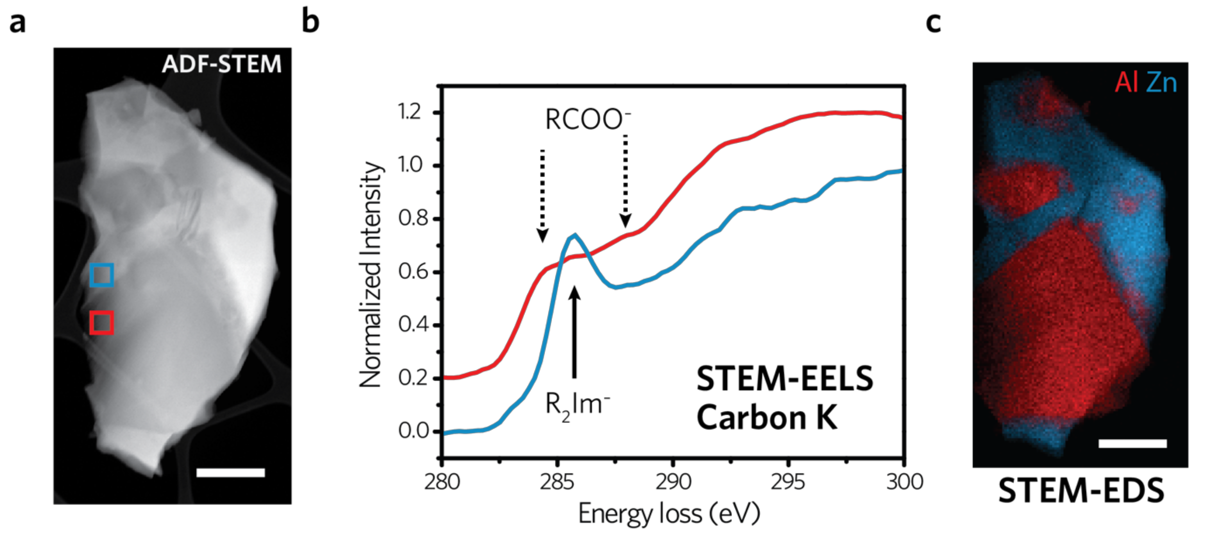

Supplementary Fig. 13. Observation of ligand chemistry by STEM-EELS of the (MIL-53)$_{0.25}$(a_gZIF-62)$_{0.75}$ CGC (thermal treatment at 450 °C for 10 min). (**a**) Annular dark field (ADF) STEM image with selected areas marked by colour-coded squares. (**b**) STEM-EELS spectra at the carbonenery *K* ionisation edge corresponding to the two selected areas in (**a**). EELS signals in the range of 284-290 eV are indicate of the carbon bonding environment due to the appearance of sharp peaks associated with chemically sensitive π* states above the Fermi energy. The carbon spectrum corresponding to the Al-rich phase (red) exhibits two low-intensity π* features at approximately 284 eV and 288 eV (marked with dashed arrows), whereas the carbon spectrum corresponding to the Zn-rich phase (blue) exhibits a single sharp π* peak at approximately 285.5 eV. The energy axis was scaled relative to the carbon *K* edge features for amorphous carbon (in the lacey carbon support film), with a π* at 284.5 eV. The sharp π* peak at 285.5 eV is characteristic of carbon *K* edge features observed in ZIF-62 and matches previous reports on the imidazole ligand [4]. The lower intensity peaks at 284 and 288 eV are consistent with reports of XANES spectra for carboxylic acid moieties [5]. (**c**) Corresponding STEM-EDS map of the Al and Zn elemental distribution. Scale bars are 400 nm.

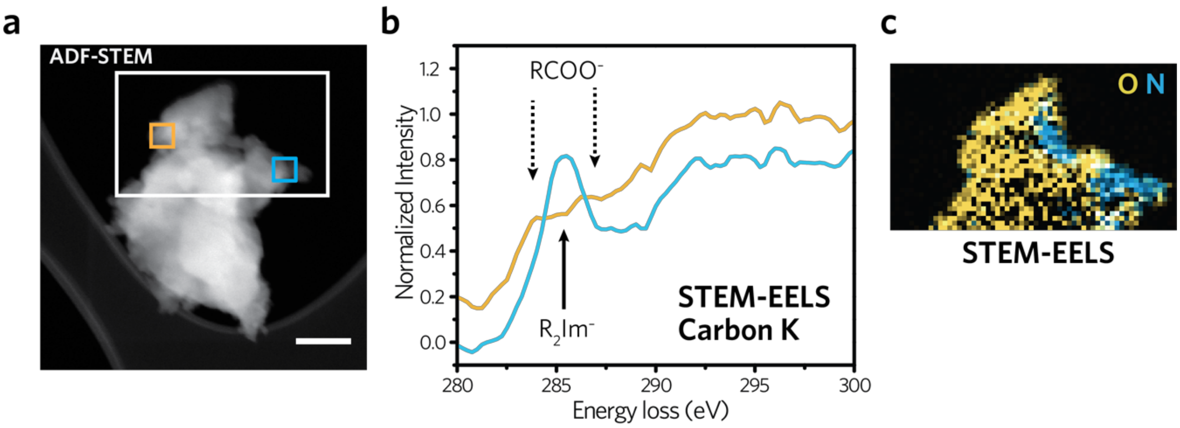

Supplementary Fig. 14. Observation of changes in ligand chemistry by STEM-EELS of the $(UiO\text{-}66)_{0.25}(a_gZIF\text{-}62)_{0.75}$ CGC composite (thermal treatment at 450 °C for 10 min). (**a**) ADF-STEM image with selected areas marked by colour-coded squares and a rectangular region of interest demarcated in orange. (**b**) STEM-EELS spectra at the carbon K ionisation edge corresponding to the two selected areas in (**a**). (**c**) Corresponding STEM-EELS map of the O and N elemental distribution. The scale bar is 100 nm.

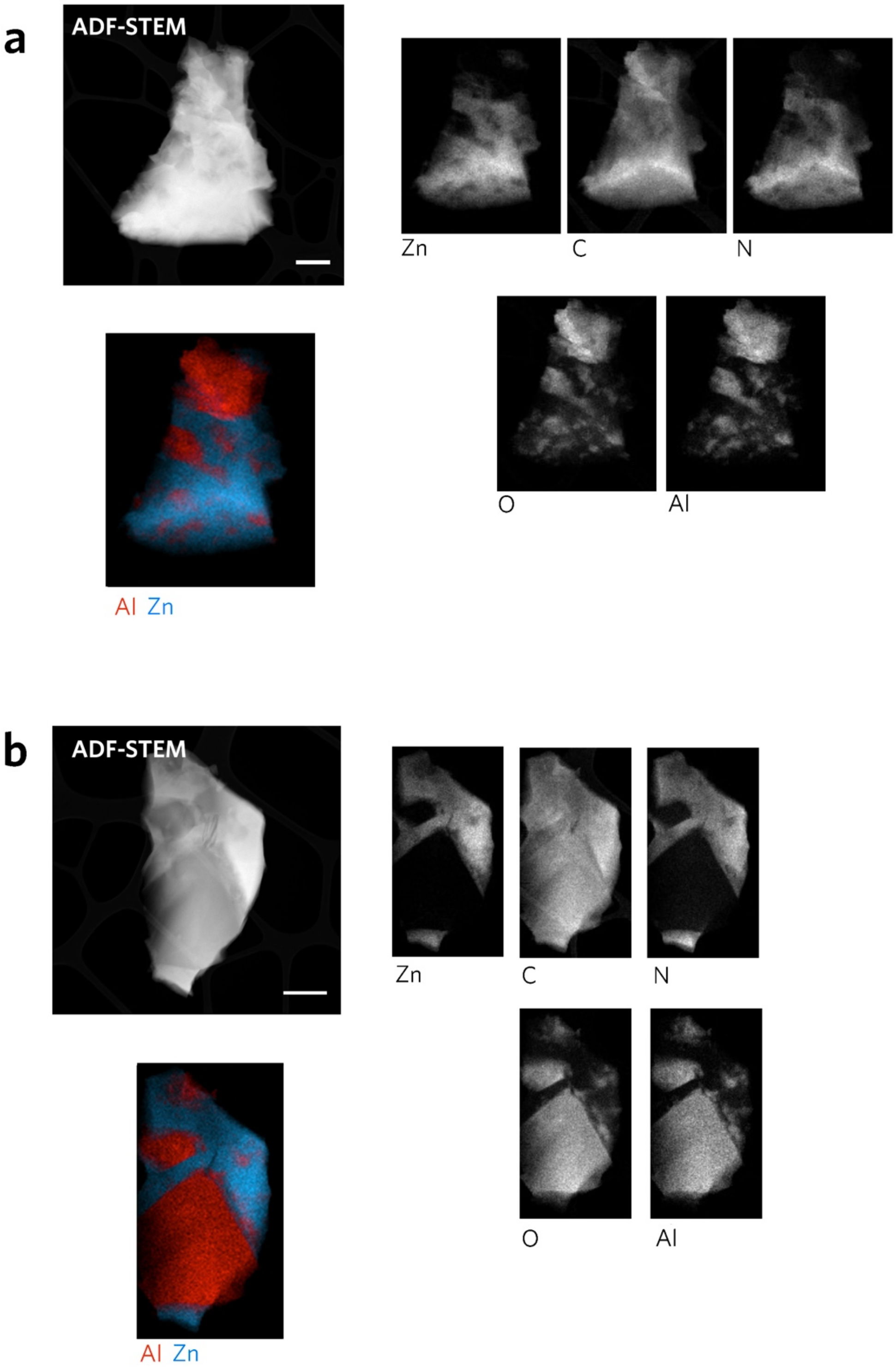

Supplementary Fig. 15. STEM micrographs combined with EDS maps of the (MIL-53)$_{0.25}$(a_gZIF-62)$_{0.75}$ CGCs. The distribution of ZIF-62 can be confirmed with the elemental mapping of Zn and N, and the distribution of MIL-53 is correlated to the elemental distribution of Al and O. Scale bars are (**a**) 500 nm and (**b**) 400 nm.

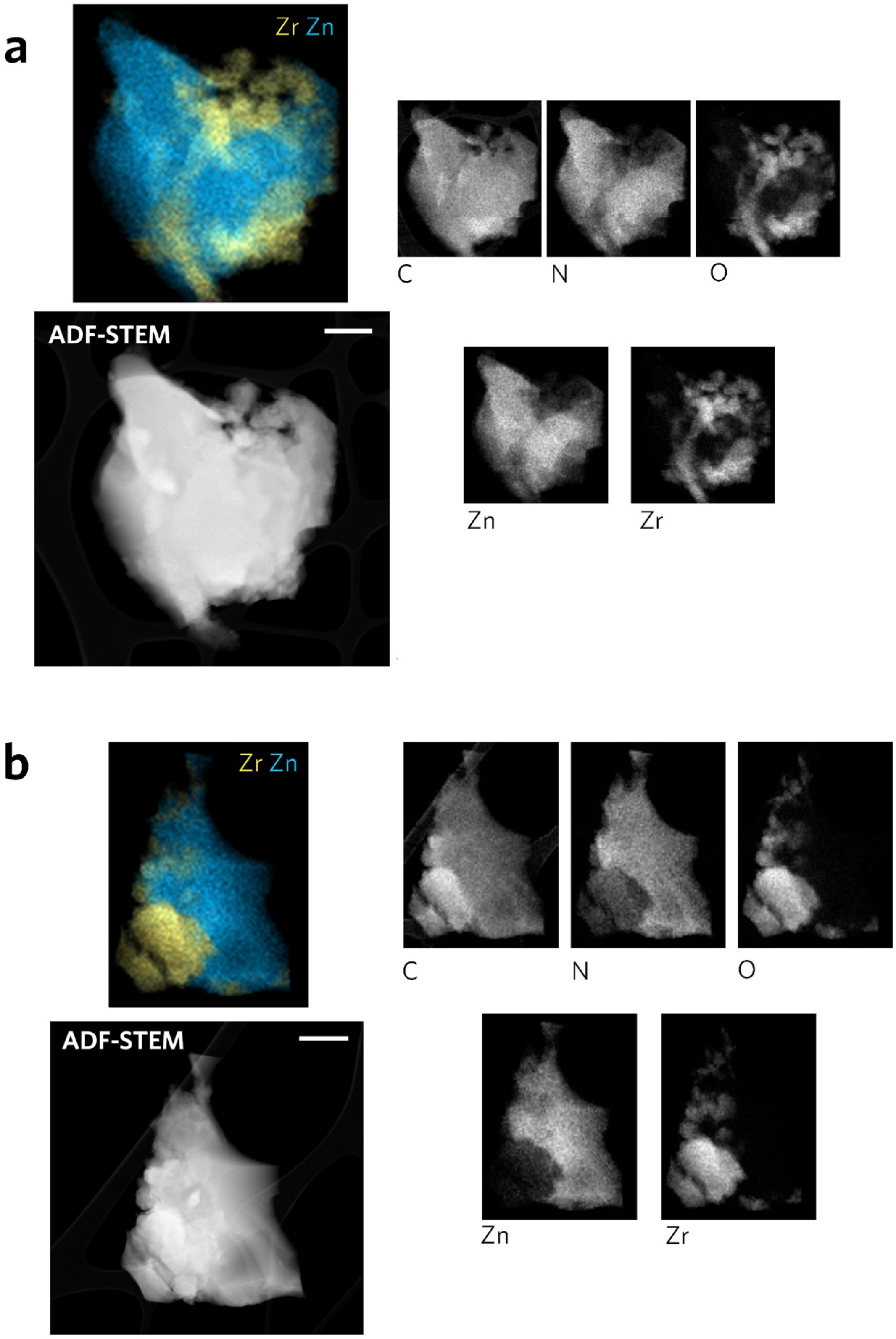

Supplementary Fig. 16. STEM micrographs combined with EDX maps of the (UiO-66)$_{0.25}$(a_gZIF-62)$_{0.75}$ CGCs. The distribution of UiO-66 can be correlated to the elemental distribution of Zr and O. Scale bars are (**a**) 300 nm and (**b**) 500 nm.

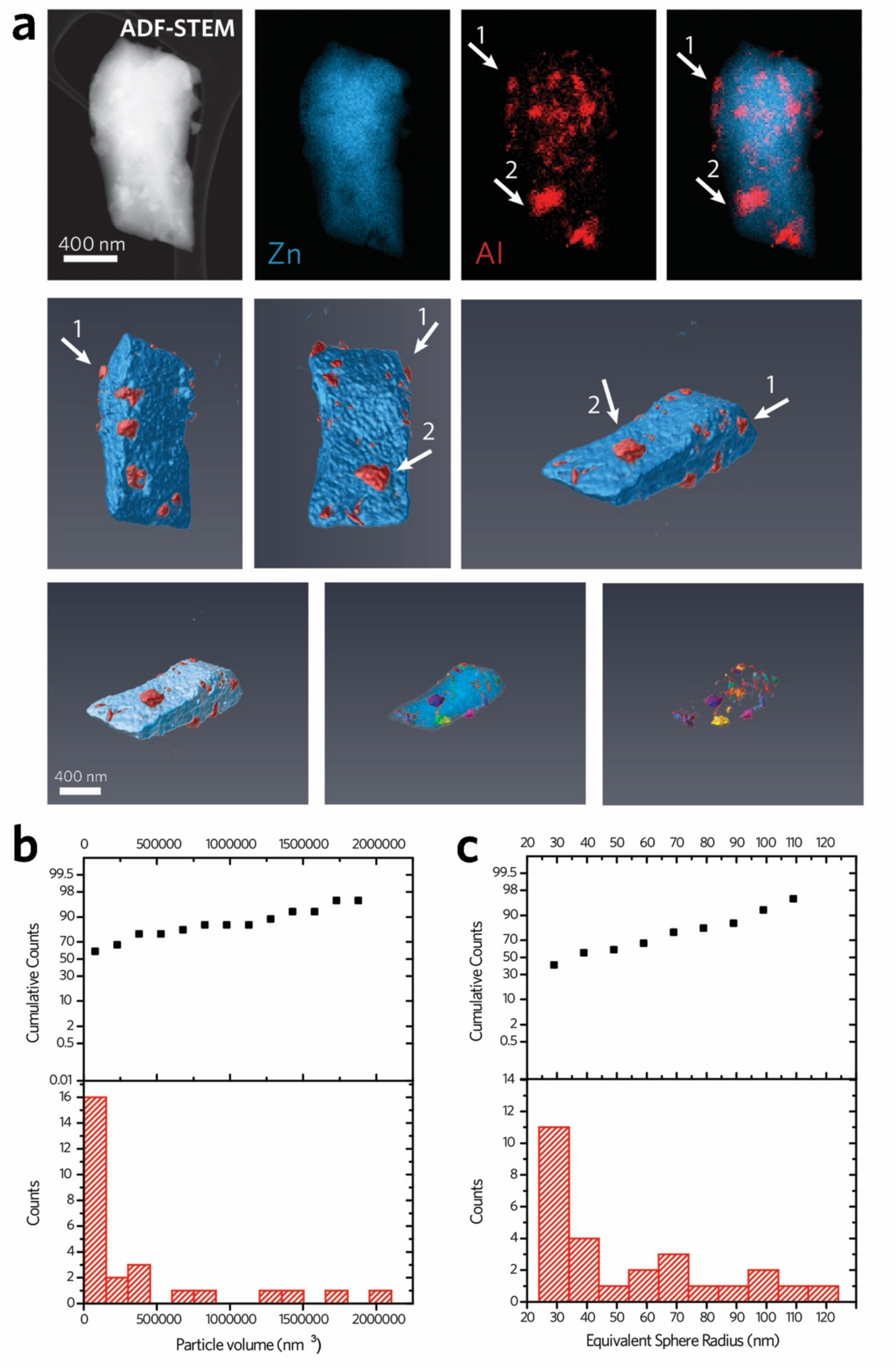

Supplementary Fig. 17. MIL-53 particle distribution and particle size analysis by tomography for the (MIL-53)$_{0.25}$(a_gZIF-62)$_{0.75}$ CGC. (**a**) 3D rendered image of the (MIL-53)$_{0.25}$(a_gZIF-62)$_{0.75}$ CGC, with MIL-53 particles identified and separated. Scale bar is 400 nm. ZIF-62 glass is indicated in blue and MIL-53 crystal particles are highlighted with multiple colour markers. White arrows highlight the region of Al in both 2D and their corresponding 3D images. (**b**) MIL-53 crystal particle volume distribution profile within the CGC. (**c**) MIL-53 crystal particle size distribution profile within the CGC. CGC sample was prepared with 10 min thermal treatment at 450 °C. For b and c, particles less than 2×2×2 pixels are excluded.

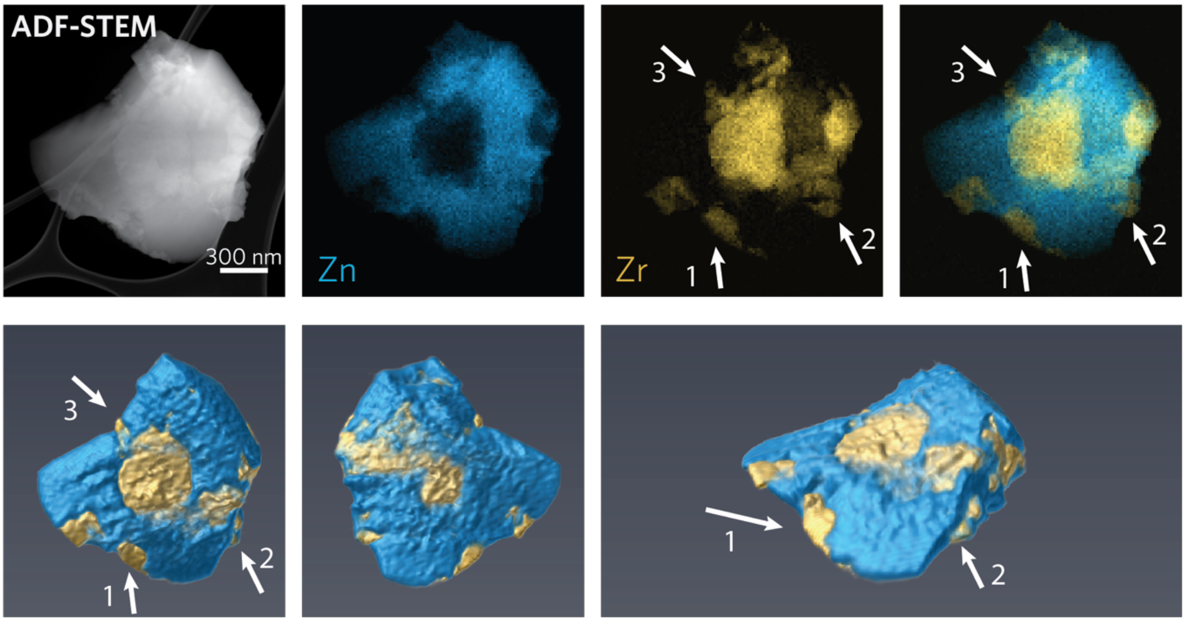

Supplementary Fig. 18. Three-dimensional tomography of $(UiO\text{-}66)_{0.25}(a_gZIF\text{-}62)_{0.75}$ CGC. The ZIF-62 glass section is indicated with blue while the UiO-66 particles are highlighted with yellow. The CGC sample was prepared with 10 min thermal treatment at 450 °C. White arrows highlight the region of Zr in both 2D and their corresponding 3D images.

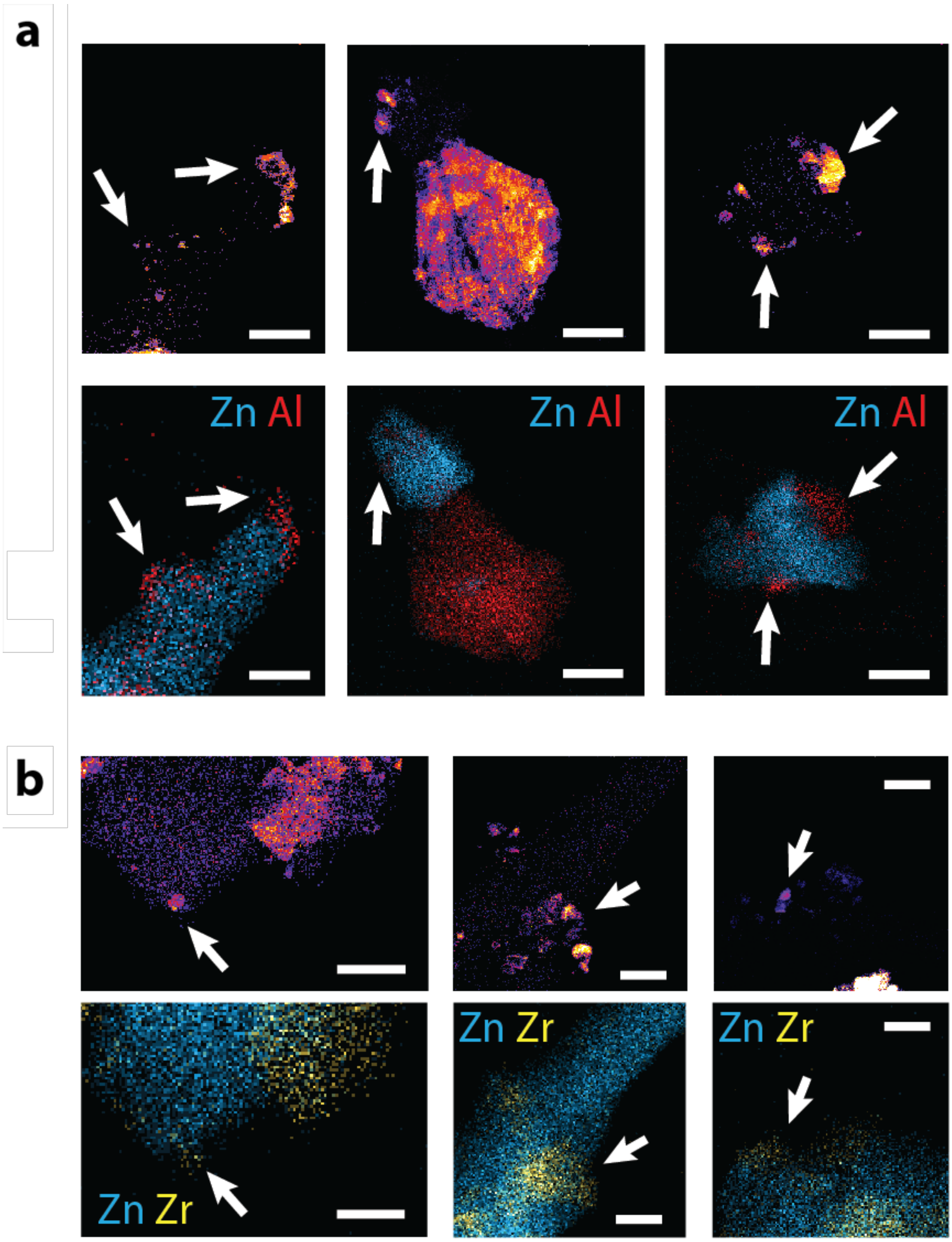

Supplementary Fig. 19. Scanning electron diffraction (SED) characterisation of the CGC samples. (**a**) STEM-SED (top row) and their corresponding STEM-EDX mapping results (bottom row) for the $(\text{MIL-53})_{0.25}(a_g\text{ZIF-62})_{0.75}$ CGC. (**b**) STEM-SED (top row) and their corresponding STEM-EDX mapping results (bottom row) for the $(\text{UiO-66})_{0.25}(a_g\text{ZIF-62})_{0.75}$ CGC. All scale bars are 200 nm. White arrows highlight the region of Al (or Zr) in both STEM-SED and their corresponding STEM-EDX mapping results.

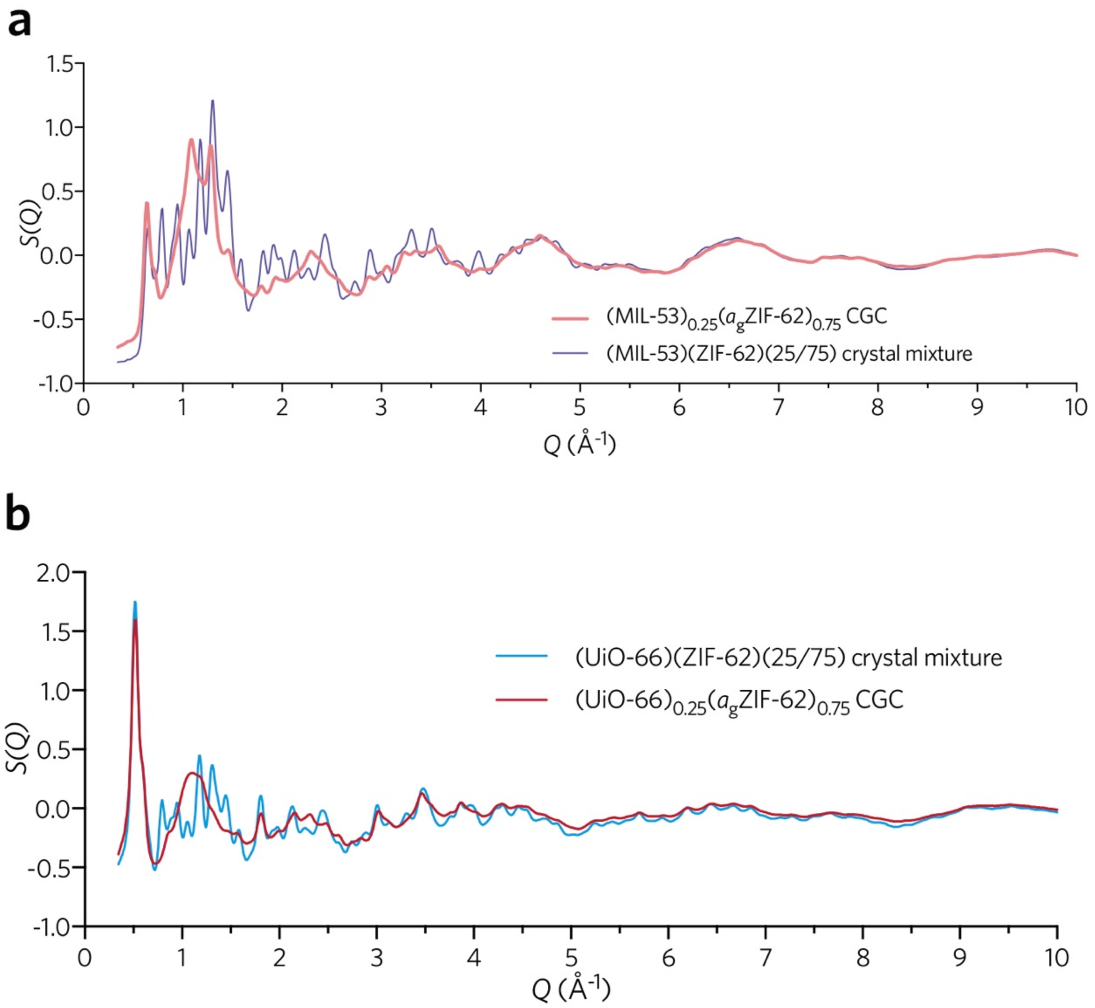

Supplementary Fig. 20. Synchrotron X-ray total scattering pattern. (a) (MIL-53)(ZIF-62)(25/75) crystal mixture and (MIL-53)$_{0.25}$(a_gZIF-62)$_{0.75}$ CGC and (**b**) (UiO-66)(ZIF-62)(25/75) crystal mixture and (UiO-66)$_{0.25}$(a_gZIF-62)$_{0.75}$ CGC.

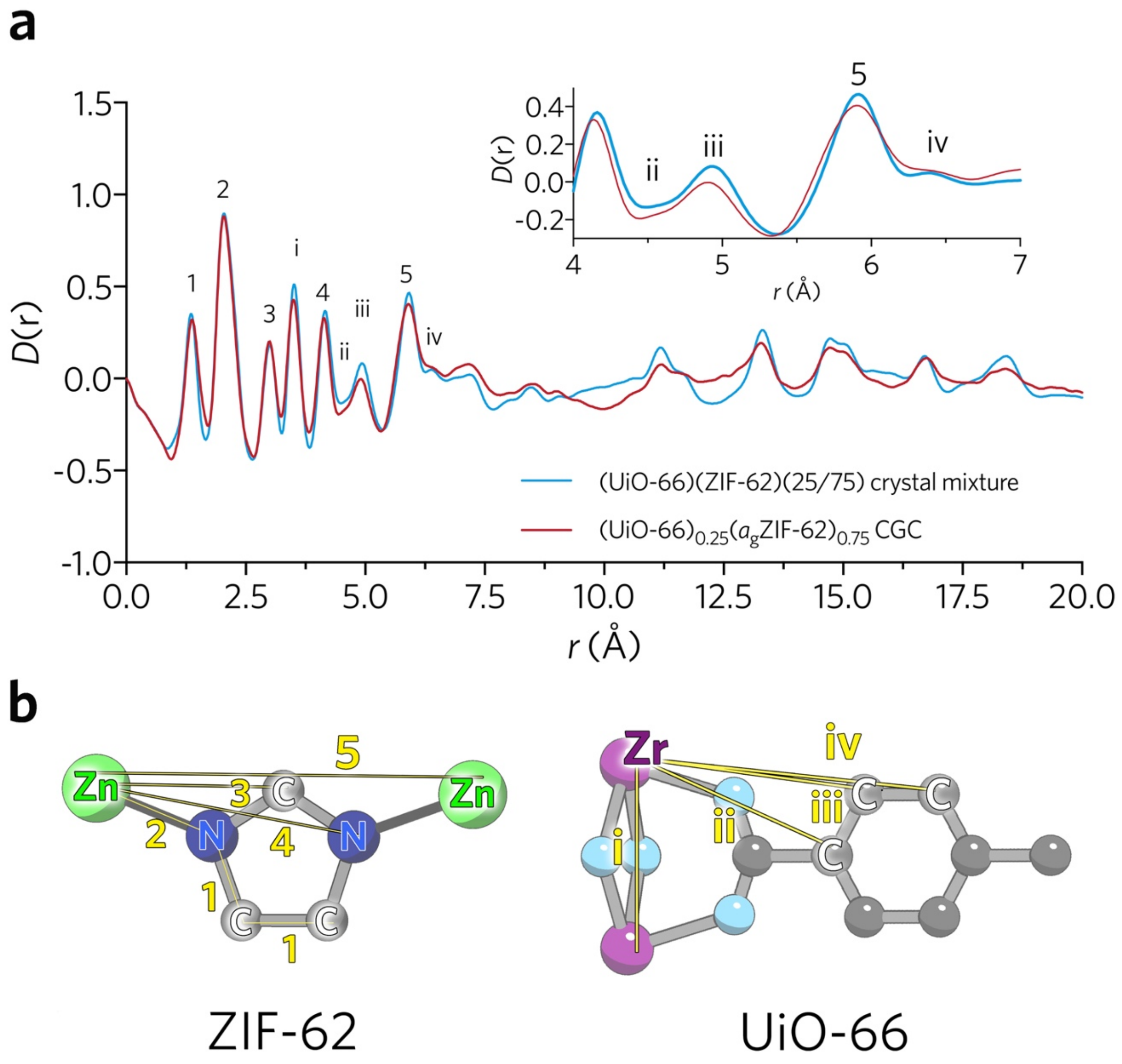

Supplementary Fig. 21. Atomic structure of the UiO-66 CGC. (**a**) Pair distribution function (PDF) *D(r)* calculated via Fourier transform of the X-ray total scattering function *S(Q)* for the (UiO-66)/(ZIF-62)(25/75) crystal mixture and (UiO-66)$_{0.25}$(a_gZIF-62)$_{0.75}$ CGC. (**b**) Scheme of peak assignment of PDF pattern.

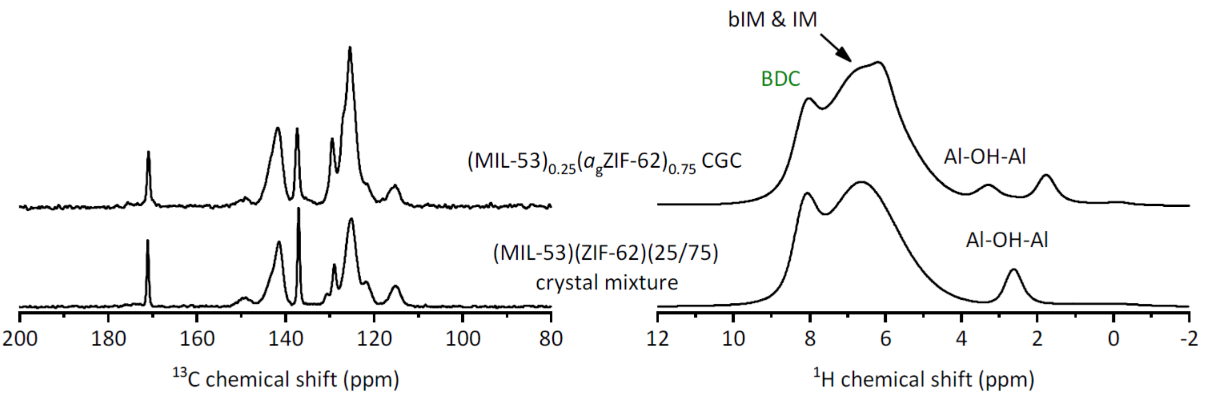

Supplementary Fig. 22. ^{13}**C (left) and** ^{1}**H (right) MAS NMR analysis for (MIL-53)(ZIF-62)(25/75) crystal mixture and (MIL-53)$_{0.25}$(a_gZIF-62)$_{0.75}$ CGC.**

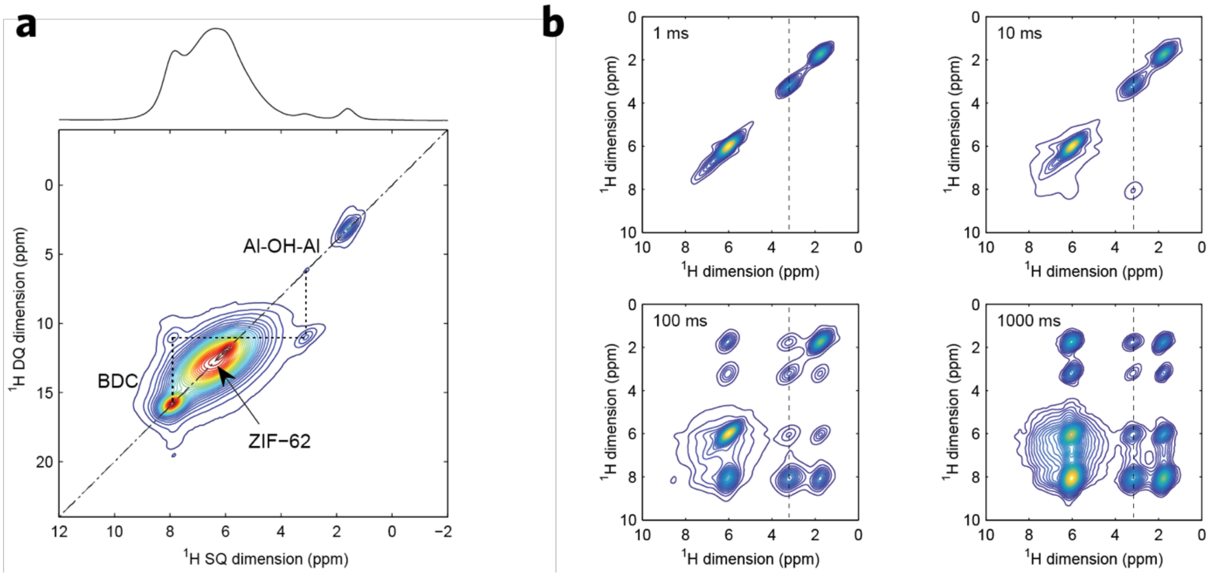

Supplementary Fig. 23. Proximities among different species within $(MIL\text{-}53)_{0.25}(a_gZIF\text{-}62)_{0.75}$ CGC as inspected by proton-proton dipolar-recoupled NMR experiments. (**a**) Two-dimensional ^1H-^1H back-to-back (BABA) spectrum, facilitating the assignment of the BDC and OH signals of MIL-53, and of the imidazolate signal of ZIF-62. (**b**) 2D ^1H-detected T_2-filtered proton-spin-diffusion spectra of the CGC sample recorded with different mixing times. Vertical dashed lines indicate the traces of the 2D spectra, which were analyzed to determine spin-diffusion curves and thus to obtain the information about the rate and extent of polarization transfer between the OH protons of MIL-53 on one hand, and BDC protons of MIL-53 and imidazolate protons of ZIF-62 on the other hand. The 2D spectra also show that species resonating at 1.7 ppm are within MIL-53 or on its surface. The CGC sample was subject to thermal treatment at 450 °C for 10 min.

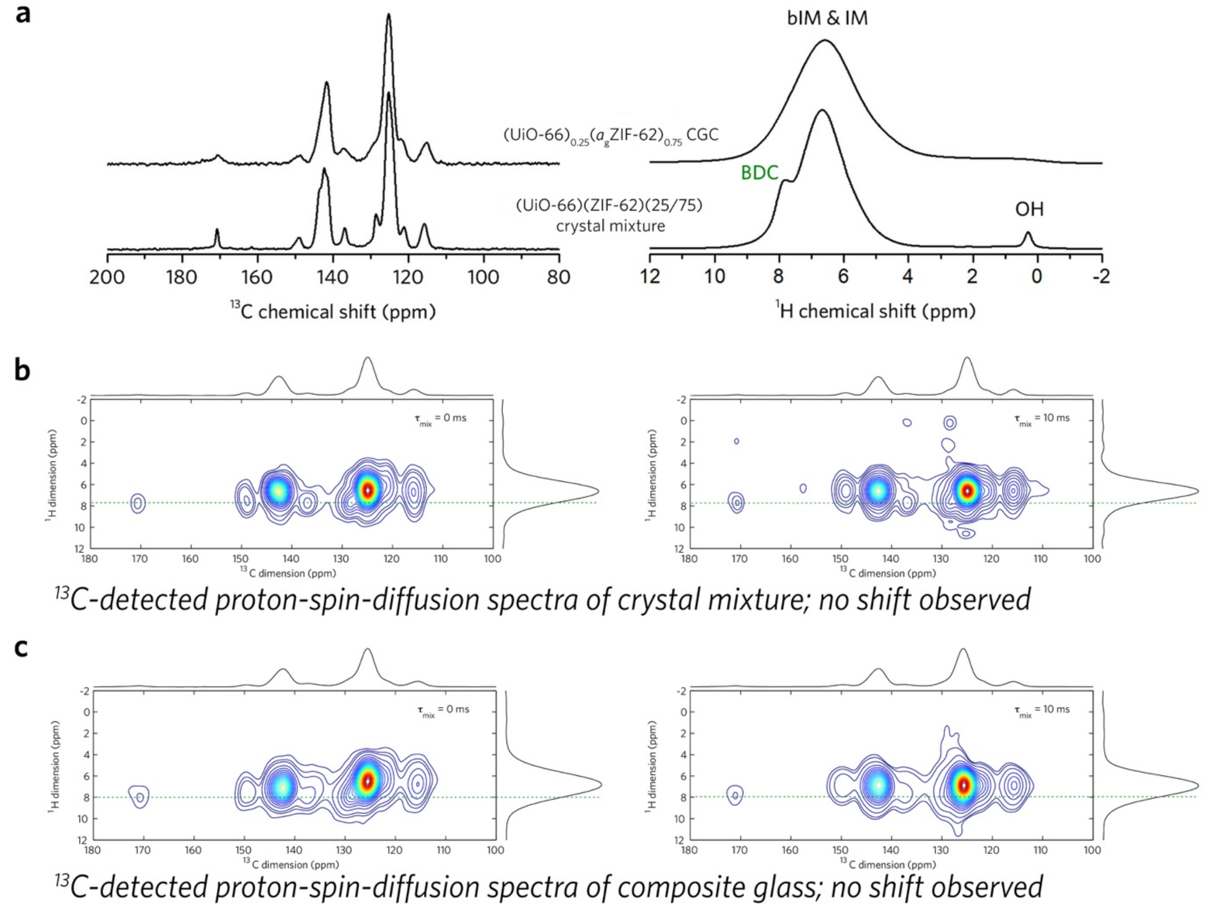

Supplementary Fig. 24. Solid-state NMR analysis of (UiO-66)(ZIF-62)(25/75) crystal mixture and (UiO-66)$_{0.25}$(a_gZIF-62)$_{0.75}$ CGC. (**a**) ^{13}C (left) and ^{1}H (right) MAS NMR spectra of crystal mixture and CGC. ^{13}C-detected proton-spin-diffusion spectra of the (**b**) crystal mixture and (**c**) CGC (left: mixing time = 0 ms, and right: mixing time = 10 ms). The CGC sample was melted by thermal treatment at 450 °C for 10 min.

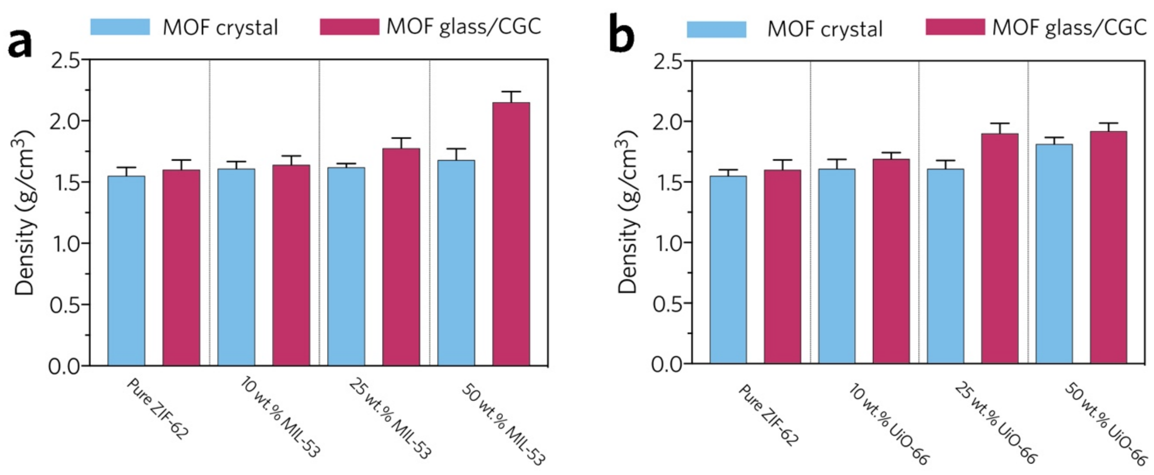

Supplementary Fig. 25. Density of MOF crystal mixture and MOF CGC. Crystal samples are in blue and glass samples are in red. S.d. is calculated based 10 measurements for each sample.

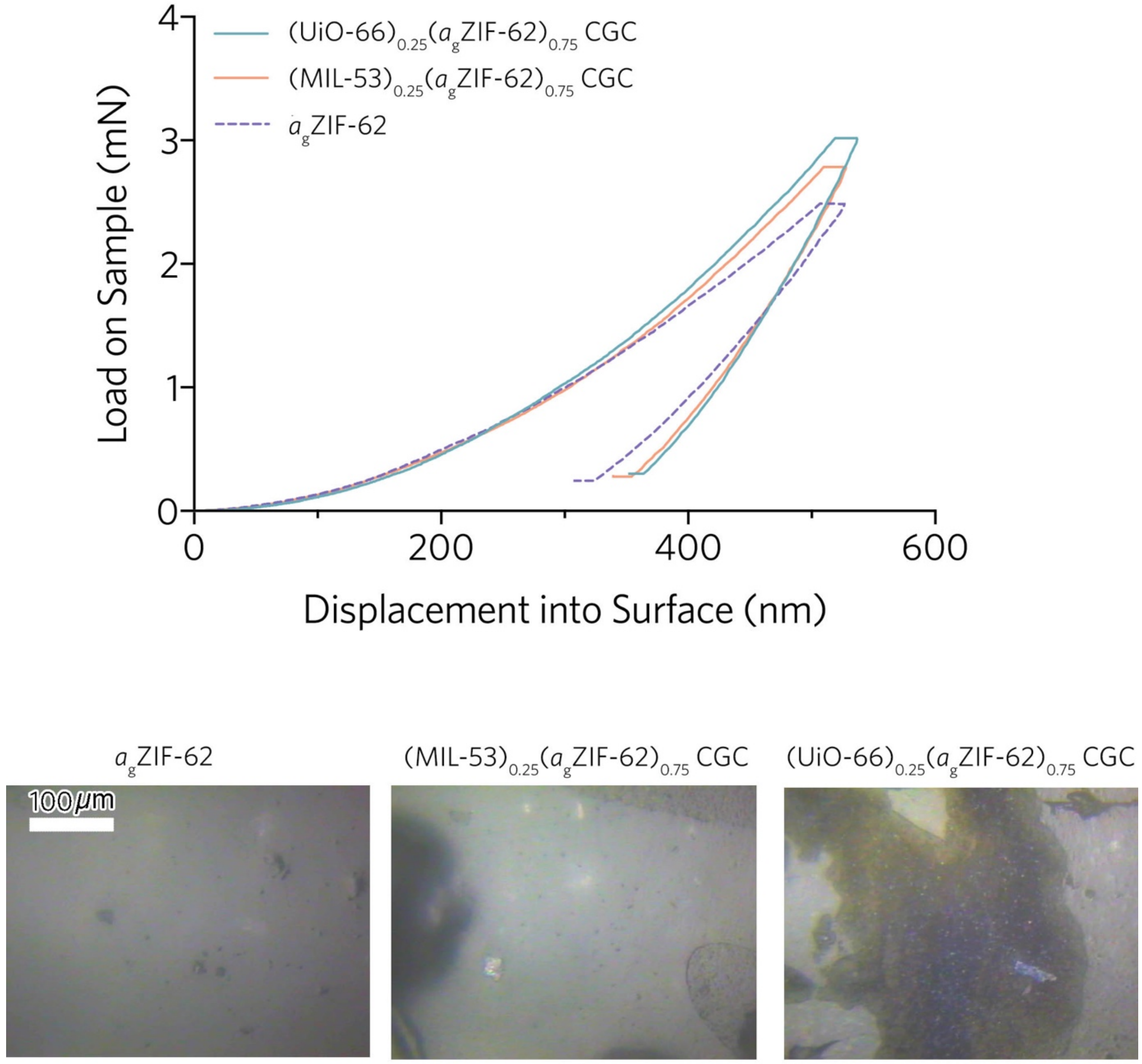

Supplementary Fig. 26. Typical load displacement data for all glass samples along with optical images of the indentations.

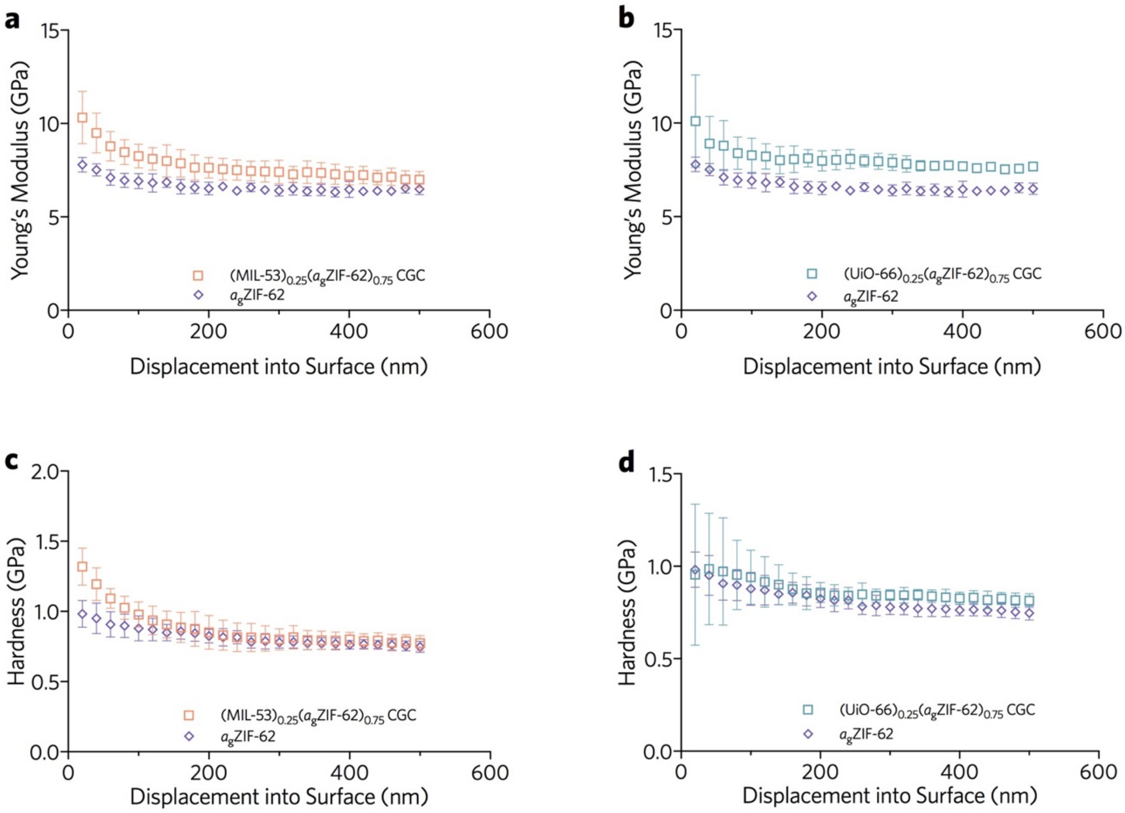

Supplementary Fig. 27. Mechanical test results for the CGCs. Variance of elastic modulus with depth for the CGCs with (**a**) MIL-53 and (**b**) UiO-66. Variance of hardness with depth for CGCs with (**c**) MIL-53 and (**d**) UiO-66. Compared with MIL-53, the larger error bars for the UiO-66 series CGC suggested the CGC is less homogeneous. S.d. is calculated based on 20 cycles of repeated tests.

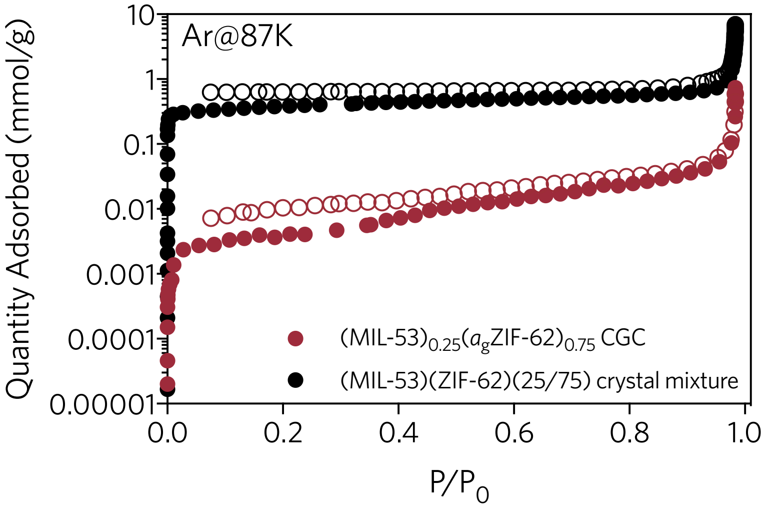

Supplementary Fig. 28. Ar adsorption (solid symbols)/desorption (open symbol) isotherms of the crystal mixture (black) and CGC (red) performed at 87 K.

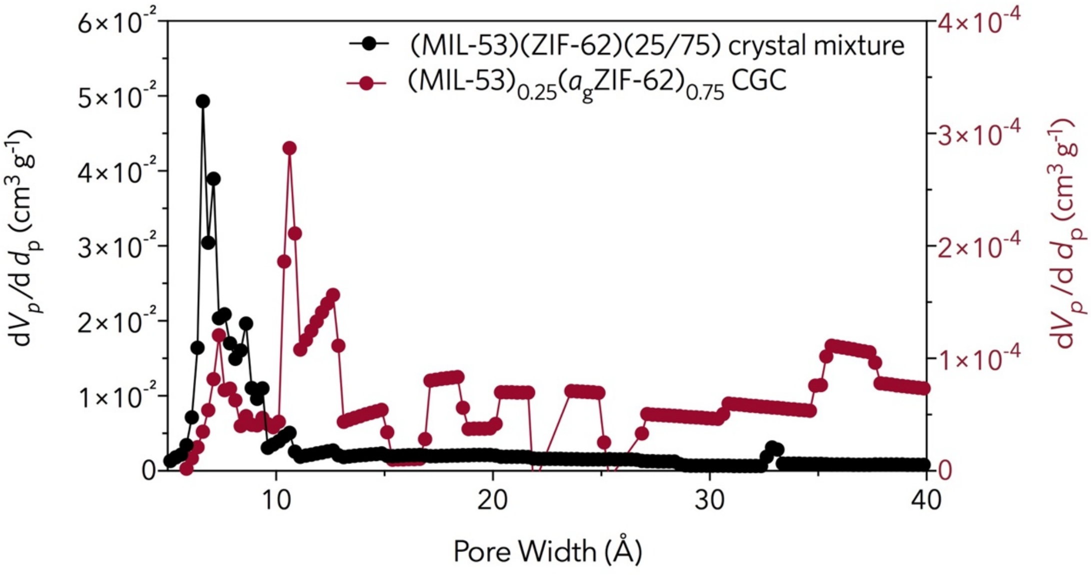

Supplementary Fig. 29. Pore size distributions for the crystal mixture (black) and CGC (red) obtained from Ar adsorption branch. The distributions were calculated using the Horwath-Kawazoe method via the Saito-Foley approach.

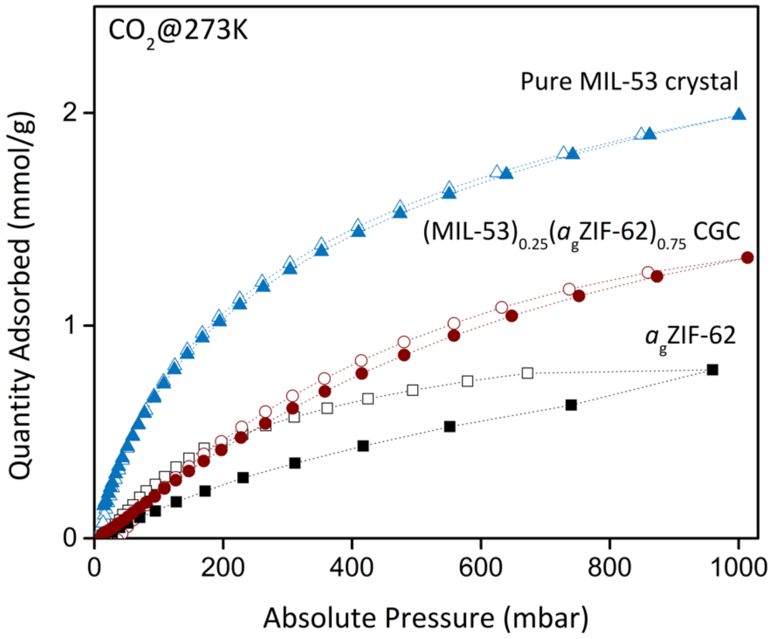

Supplementary Fig. 30. CO_2 adsorption(solid) /desorption(open) profiles at 273 K.

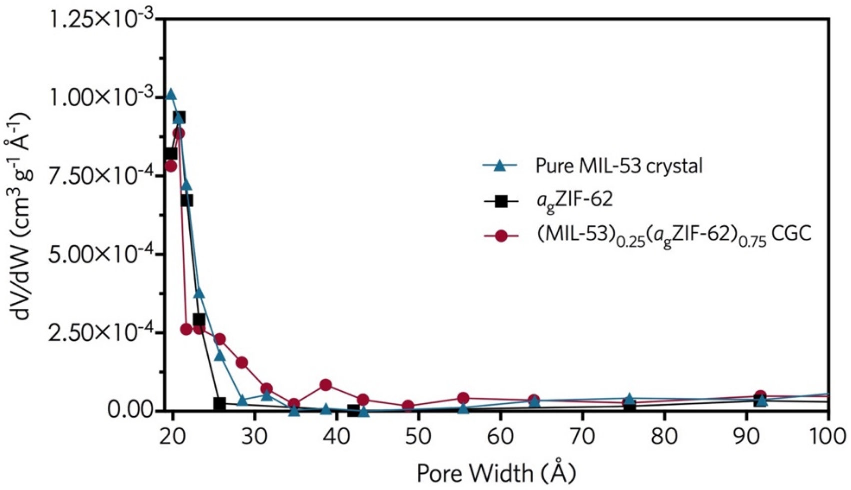

Supplementary Fig. 31. Pore size distribution from N_2 measurements at 77K, of the MOF glass and CGC in the mesoporous range. The distributions were calculated using the Barrett, Joyner, and Halenda method.

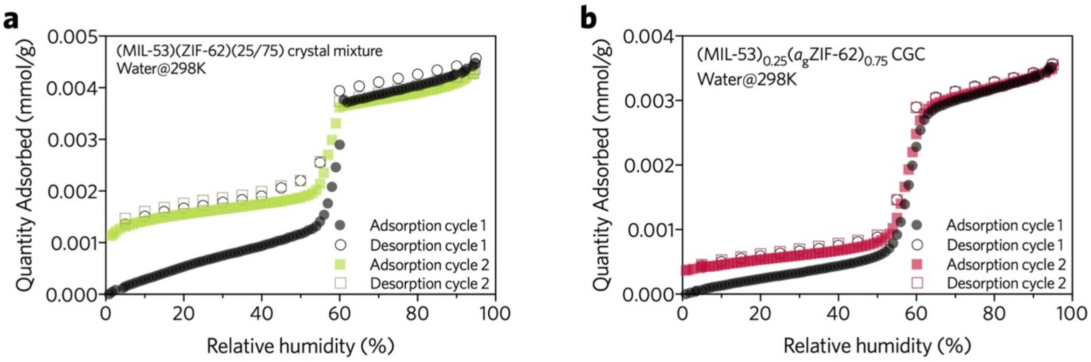

Supplementary Fig. 32. Water adsorption (solid)/desorption (open) isotherms of the (a) crystal mixture and (b) CGCs performed at 298 K.

Supplementary Table 1. Experimental and crystallographic details based on the *in-situ* synchrotron powder diffraction and *ex-situ* XRD results.*

Sample, phase and analysis conditions	(MIL-53/ZIF-62)(25/75)<br>MIL-53-as (25°C)<br>*in-situ* synchrotron powder diffraction	$(MIL\text{-}53)_{0.25}$ $(a_gZIF\text{-}62)_{0.75}$<br>MIL-53-lp (460°C)<br>*in-situ* synchrotron powder	$(MIL\text{-}53)_{0.25}$ $(a_gZIF\text{-}62)_{0.75}$<br>MIL-53-lp (RT)<br>*ex-situ* XRD	(MIL-53/ZIF-62)(25/75)<br>ZIF-62 (25°C)<br>*In-situ* synchrotron powder diffraction	Pure ZIF-62 (25°C)<br>*In-situ* synchrotron powder diffraction
Cell setting	Orthorhombic	Orthorhombic	Orthorhombic	Orthorhombic	Orthorhombic
Space group	Pnma (no.62)	Imma (no.74)	Imma (no.74)	Pbca (no. 61)	Pbca (no. 61)
a, b, c (Å)	17.115(1)<br>6.626(6)<br>12.204(9)	6.624(7)<br>16.855(0)<br>12.631(7)	6.631(6)<br>16.861(7)<br>12.645(5)	15.660(7)<br>15.642(2)<br>18.272(5)	15.660(4)<br>15.549(4)<br>18.231(0)
α, β, γ (°)	90, 90, 90	90, 90, 90	90, 90, 90	90, 90, 90	90, 90, 90
Volume ($Å^3$)	1380.2(1)	1409.3(5)	1413.7(8)	4466.7(9)	4461.2(0)
R factors and goodness-of-fit	R_p=1.04,<br>R_{wp}=1.21,<br>R_{exp}=0.95,<br>GOF=1.15	R_p=1.25,<br>R_{wp}=1.94,<br>R_{exp}=1.31,<br>GOF=1.64	R_p=1.51,<br>R_{wp}=1.27,<br>R_{exp}=1.05,<br>GOF=1.01	R_p=1.13,<br>R_{wp}=1.35,<br>R_{exp}=0.97,<br>GOF=1.15	R_p=1.25,<br>R_{wp}=1.15,<br>R_{exp}=1.21,<br>GOF=1.01

*Pawley refinements were carried out with TOPAS Commercial V4.0 software package with the published cif files [1,2]

Reference

1. Loiseau, T. *et al.* A Rationale for the Large Breathing of the Porous Aluminum Terephthalate (MIL-53) Upon Hydration. *Chem.: Eur. J.* **10**, 1373–1382 (2004).
2. Banerjee, R. *et al.* High-Throughput Synthesis of Zeolitic Imidazolate Frameworks and Application to CO_2 Capture. *Science* **319**, 939–943 (2008).
3. Valenzano, L. *et al.* Disclosing the Complex Structure of UiO-66 Metal Organic Framework: A Synergic Combination of Experiment and Theory. *Chem. Mater.* **23**, 1700–1718 (2011).
4. Apen, E., Hitchcock, A. P. & Gland, J. L. Experimental Studies of the Core Excitation of Imidazole, 4,5-dicyanoimidazole, and s-triazine. *J. Phys. Chem.* **97**, 6859–6866 (1993).
5. Urquhart, S. G. & Ade, H. Trends in the Carbonyl Core (C 1S, O 1S) → $\pi^*_{C=O}$ Transition in the Near-Edge X-ray Absorption Fine Structure Spectra of Organic Molecules. *J. Phys. Chem. B* **106**, 8531–8538 (2002).
