# Supplementary material for: Metal-organic framework crystal-glass composites
Source: Nat Commun. 2019 Jun 12;10:2580. doi: 10.1038/s41467-019-10470-z (PMC6561910; doi:10.1038/s41467-019-10470-z)
Supplement: Supplementary file 2 — Peer Review File [file 41467_2019_10470_MOESM2_ESM.pdf]

Editorial Note: This manuscript has been previously reviewed at another journal. The manuscript was considered suitable for publication without further review at Nature Communications.
